# Supplementary material for: The Anterior Cingulate Cortex Predicts Future States to Mediate Model-Based Action Selection
Source: Neuron. 2021 Jan 6;109(1):149–163.e7. doi: 10.1016/j.neuron.2020.10.013 (PMC7837117; doi:10.1016/j.neuron.2020.10.013)
Supplement: Document S1. Figures S1–S8 [file mmc1.pdf]

Neuron, Volume *109*

Supplemental Information

The Anterior Cingulate Cortex Predicts Future States to Mediate Model-Based Action Selection

Thomas Akam, Ines Rodrigues-Vaz, Ivo Marcelo, Xiangyu Zhang, Michael Pereira, Rodrigo Freire Oliveira, Peter Dayan, and Rui M. Costa

Supplementary figures:

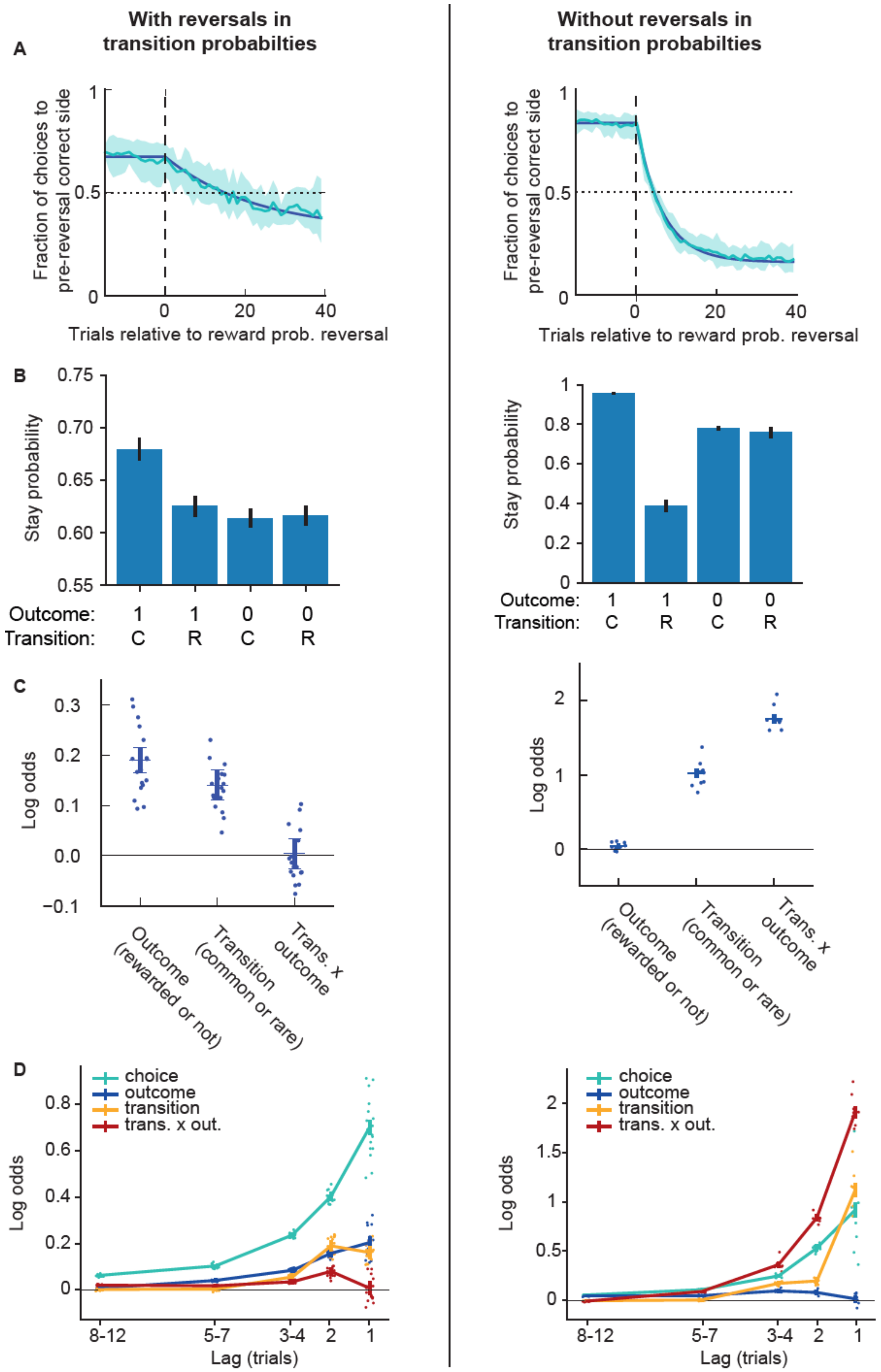

Figure S1 Behaviour without transition probability reversals, related to figures 1 & 2. The two-step task reported in the main results included reversals in the transition probability mapping the first-step actions to the second-step states, because without them, extensively trained animals could in principle learn strategies

that look like model-based RL but in fact rely on latent state inference rather than planning (Akam et al., 2015, PLOS Comp. Biol., 2015, *11*, e1004648). To assess what impact dynamically changing transition probabilities had on behaviour, we ran a version of the task where the transition probabilities linking the first step actions to second-step states were fixed (n=10 mice, 240 sessions analysed from day 22+ of training). Here we compare behaviour on the versions with transition probability reversals (left panels – reproduced from figures 1 and 2 for ease of comparison) and without transition probability reversals (right panels). The tasks were identical apart from the presence/absence of transition probability reversals. **A)** Choice probability trajectories around reward probability reversals. Pale blue line – average trajectory, dark blue line – exponential fit, shaded area – cross-subject standard deviation. Subjects were much better at tracking the correct option on the fixed task, choosing it at the end of blocks on 0.83 ± 0.04 (mean + SD) of trials, and adapting to reversals with a time constant of 6.5 trials (P<0.001 for difference between tasks on both measures, permutation test). Note that fixing the transition probabilities does not change the contrast between good and bad choices in terms of their reward probabilities. **B)** Stay probability analysis showing the fraction of trials the subject repeated the same choice following each combination of trial outcome (rewarded (1) or not (0)) and transition (common (C) or rare (R)). Error bars show cross-subject SEM. **C)** Logistic regression model fit predicting choice as a function of the previous trial's events. Predictor loadings plotted are; *outcome* (repeat choices following rewards), *transition* (repeat choices following common transitions) and *transition-outcome interaction* (repeat choices following rewarded common transition trials and non-rewarded rare transition trials). Error bars indicate 95% confidence intervals on the population mean, dots indicate maximum a posteriori (MAP) subject fits. The granular structure of behaviour was very different on the fixed task, with a very strong influence of the transition-outcome interaction on the subsequent choice (P<0.001, bootstrap test), a strong influence of the state transition (P<0.001), but no direct influence of the trial outcome (P=0.42) (between task differences at trial -1: P<0.001 for stronger loading on transition and transition-outcome interaction predictor, P=0.031 for weaker loading on outcome, permutation test). **D)** Lagged logistic regression model predicting choice as a function of events over the previous 12 trials. Predictors are as in **C**, predictor loading at lag x indicates the influence of events at trial t on choice at trial $t + x$. Overall, these data show that in the fixed task, where subjects can, in principle, learn habit-like mappings from where rewards have recently been obtained to the correct first-step action (e.g. rewards on the left →choose up), overall performance was higher and behaviour showed a strong transition-outcome interaction, which can be generated by model-based RL or latent state inference based strategies (Akam et al., 2015). The striking differences between behaviour on the fixed task and the version with transition reversals suggest that subjects do indeed solve them using different strategies.

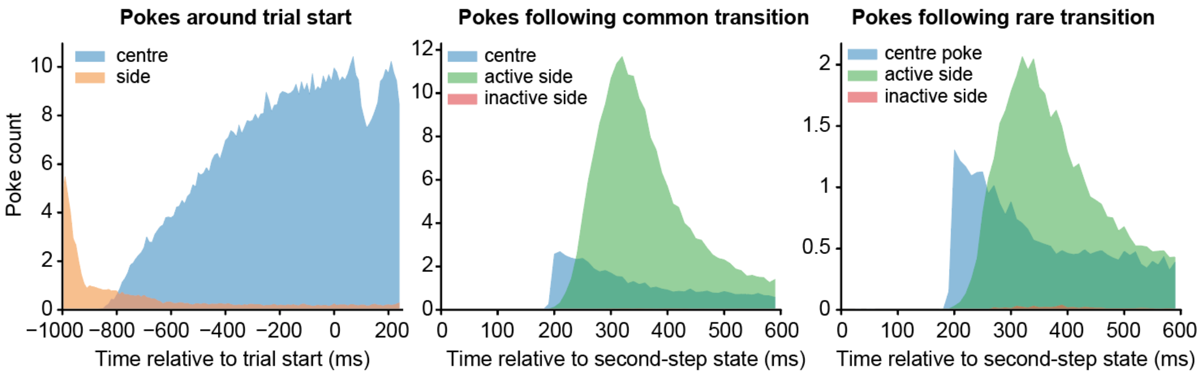

Figure S2 Poke timings around trial events, related to figure 1 A) Histogram showing the timing of pokes to the centre (i.e. top and bottom) ports (blue) and side (i.e. left and right) ports (orange) relative to the start of the trial, i.e. relative to when the centre ports become active. Subjects poke the centre ports at an increasing rate over the 1 second ITI preceding the trial start, but very rarely poke the side ports. **B)** Histogram showing the timing of pokes to the centre ports (blue) and the active (green) and inactive (red) side ports relative to the time the second-step state was entered following a common transition. The 'active' side port is the port corresponding to the second-step state reached on the trial, e.g. if the subject reached the 'left-active' state then the left port is the active side port and the right port is the inactive side port. Subjects very rarely poked the inactive side port but sometimes did an additional poke to the central ports before poking the active side. **C)** As **B** but following rare transitions.

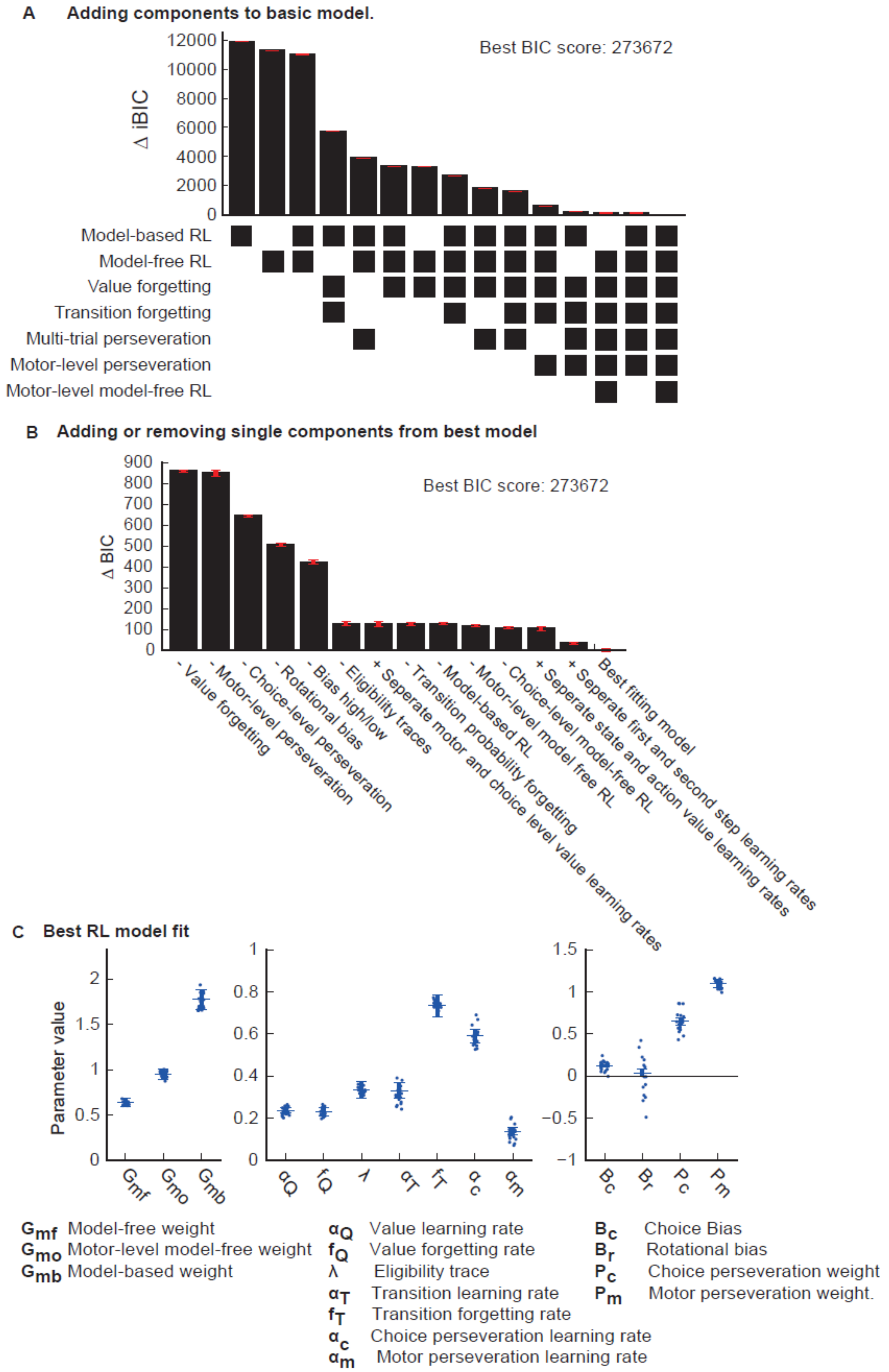

Figure S3 RL model comparison and fit, related to figure 2. A-B) RL model comparison on the baseline behavioural dataset using iBIC scores. The set of models shown in **A** were constructed by adding features to a basic model (see below). The grid below the plot indicates which features were included in each model. The set of models shown in **B** were constructed by adding or removing one feature from the best fitting model. Error-

bars indicate the bootstrap 95% confidence interval on the BIC score. **C)** Parameter values for best fitting RL model. Bars indicate 95% confidence intervals on the population mean, dots indicate maximum a posteriori (MAP) subject fits. The starting point for model comparison was the RL agent used in the original two-step task. As transition probabilities in our task are not fixed, this was modified to learn action-state transition probabilities online from experienced transitions. We also added a 'rotational' bias to move clockwise/counter-clockwise around the set of pokes (e.g. left→top, right→bottom), which we had observed in some subjects. This bias may have developed because it is the simplest fixed response pattern that was not penalised by the block transition rule (block transitions depended on behaviour, so a bias for top/bottom resulted in the preferred port spending more the time as the bad option). Using this basic model, the mixture agent incorporating both model-based and model-free RL fit better than either the pure model-free (ΔiBIC=264) or pure model-based agent (ΔiBIC=888). However, an exploratory process of model comparison indicated several additional features of the behaviour that substantially improved fit quality. The first was forgetting about actions not taken and states not visited. Value forgetting, implemented as value decay towards zero (Ito and Doya, 2009, J. Neurosci. *29*, 9861–9874), produced a dramatic improvement in fit (ΔiBIC=7698 for mixture model). Forgetting about action-state transition probabilities, implemented as decay towards a uniform distribution, further improved fit (ΔiBIC=643). The second feature found to improve fit quality was multi-trial perseveration, i.e. a tendency to repeat choices that extended over multiple trials (Akaishi et al. 2014, Neuron *81*, 195–206). Implemented as an exponential choice kernel, this provided a further substantial improvement in fit quality (ΔiBIC=1057). The final features found to improve fit were perseveration and model-free RL operating at the level of motor actions (e.g. left→top) in addition to at the level of choices (top vs bottom). Motor perseveration, implemented by maintaining separate exponential choice kernels for trials that ended on the left and right, which each influenced choices following trials ending on their respective sides, substantially improved fit quality (ΔiBIC=1503). Model-free value learning at the level of motor actions (in addition to choices) further improved goodness of fit (ΔiBIC=117). A similar finding has been reported in human two-step task behaviour where model-free value accrues to low level, task irrelevant, sensory-motor features (Shahar et al., 2019, PNAS *116*, 15871–15876). With all these additional features added to the model, the mixture agent still provided a better fit to the data than either a pure model-free (ΔiBIC=127) or pure model-based (ΔiBIC=227) agent. We tested a number of other modifications to the model including separate learning rates at the first and second step, but did not find further improvements in fit quality (see **B**). Finally, as adding features may make other features which previously improved the fit unnecessary, we tested whether removing any individual component from the model improved fit quality but again did not find further improvements (see **B**). For a more extensive discussion of the RL model-comparison, see the Supplementary Results section of the bioRxiv version of this paper (https://doi.org/10.1101/126292).

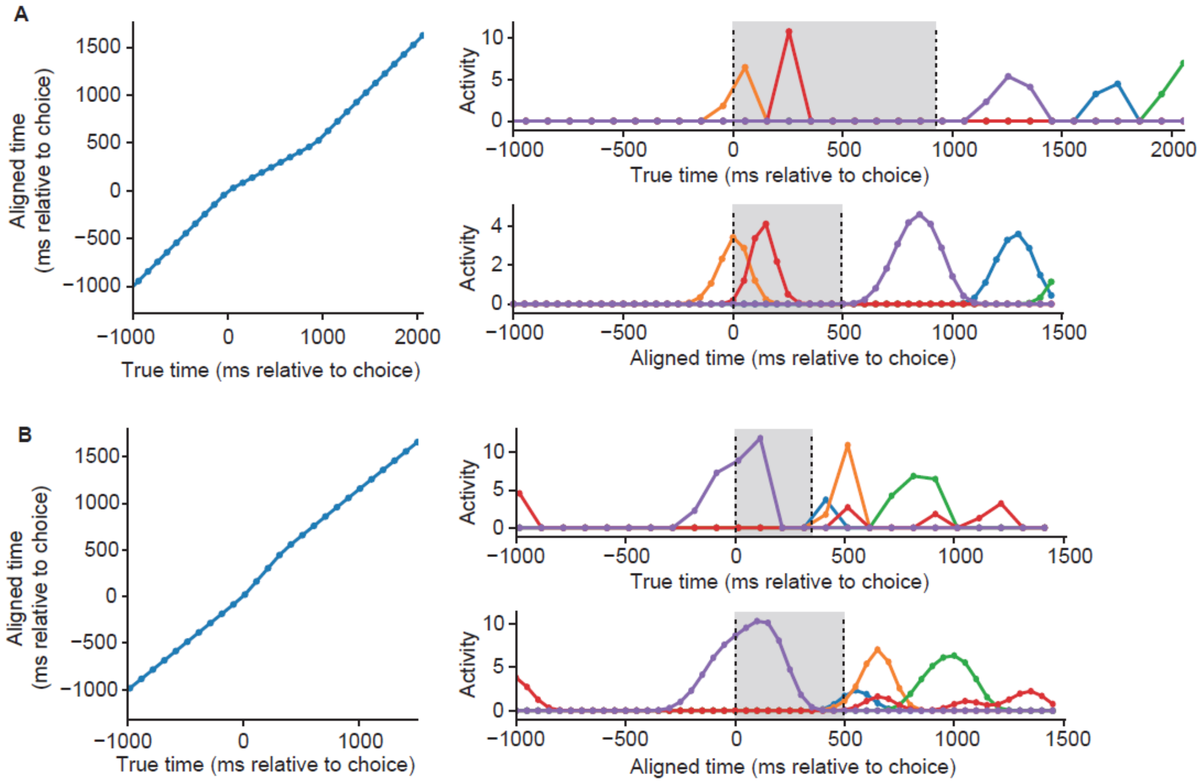

Figure S4. Calcium imaging alignment, up-sampling and smoothing, related to figure 3 & STAR methods. A) Alignment of imaging data on a trial where the interval between choice and second-step port entry was longer than the median interval. Left panel shows the true and aligned times of microscope frames plotted against each other. Right top panel shows the activity of 5 neurons before alignment. Vertical dashed lines show the times of choice and second-step port entry. Right bottom panel shows the activity of the same 5 neurons after alignment, up-sampling and smoothing. Grey shaded regions indicate the interval between choice and second-step port entry that is time-warped **B)** As for **A** but for a trial where the interval between choice and second-step port entry was shorter than the median interval.

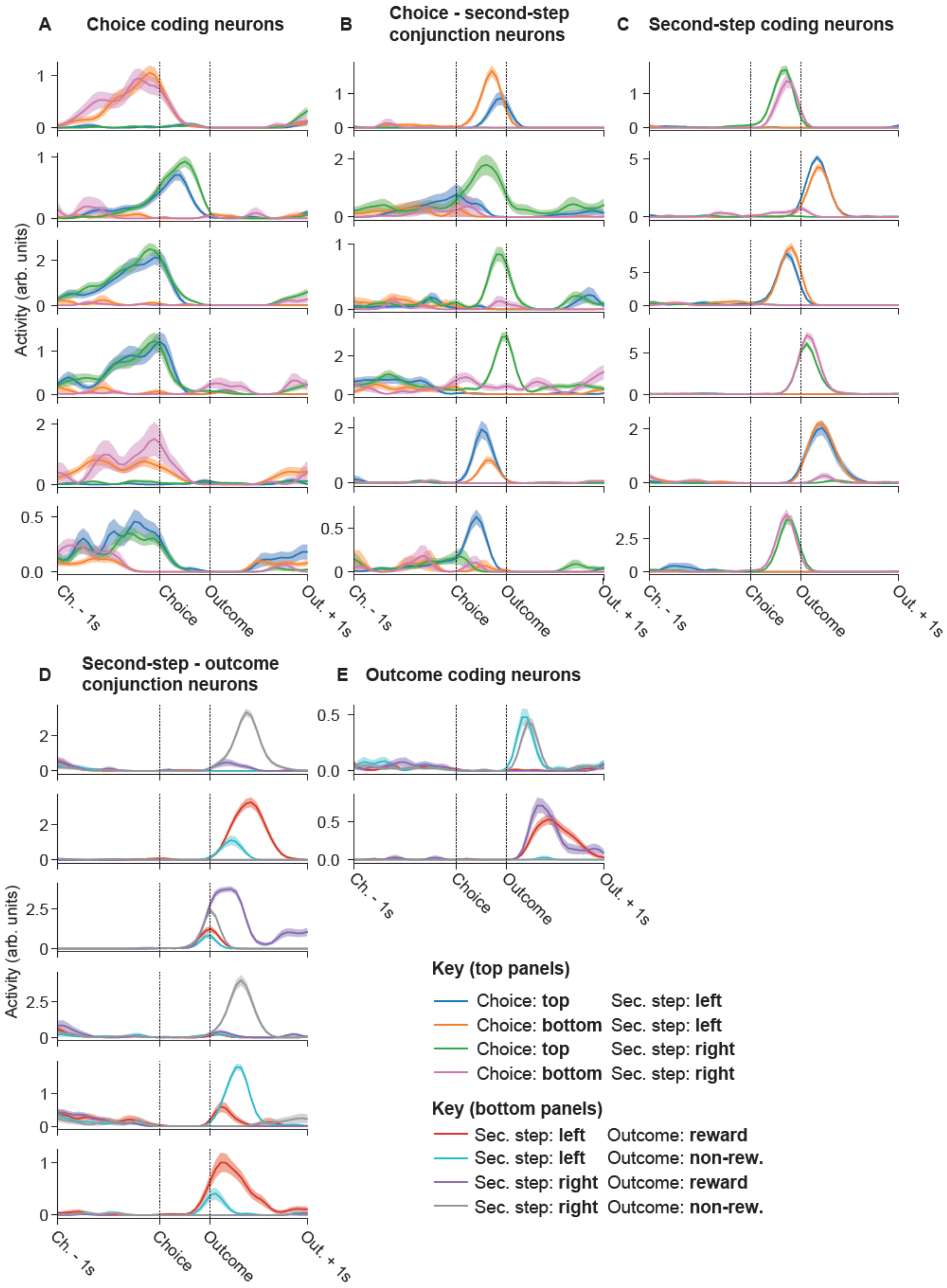

Figure S5 Example neuron activity, related to figure 3. A) Activity for six example neurons which coded the subjects choice. Traces show average activity across the trial for each of 4 conditions defined by the choice and second-step state reached, shaded areas show cross-trial standard error. **B)** As **A** but for neurons which were tuned to particular conjunctions of choice and second-step state. **C)** As **A** but for neurons which coded second-step state. **D)** As **A** but for neurons which were tuned to particular conjunctions of second-step state and outcome. Trials were split by second-step state and outcome. **E)** As **D** but for neurons which were tuned to trial

outcome. Only 2 neurons are shown due to the difficulty of finding neurons strongly tuned to outcome irrespective of second-step state.

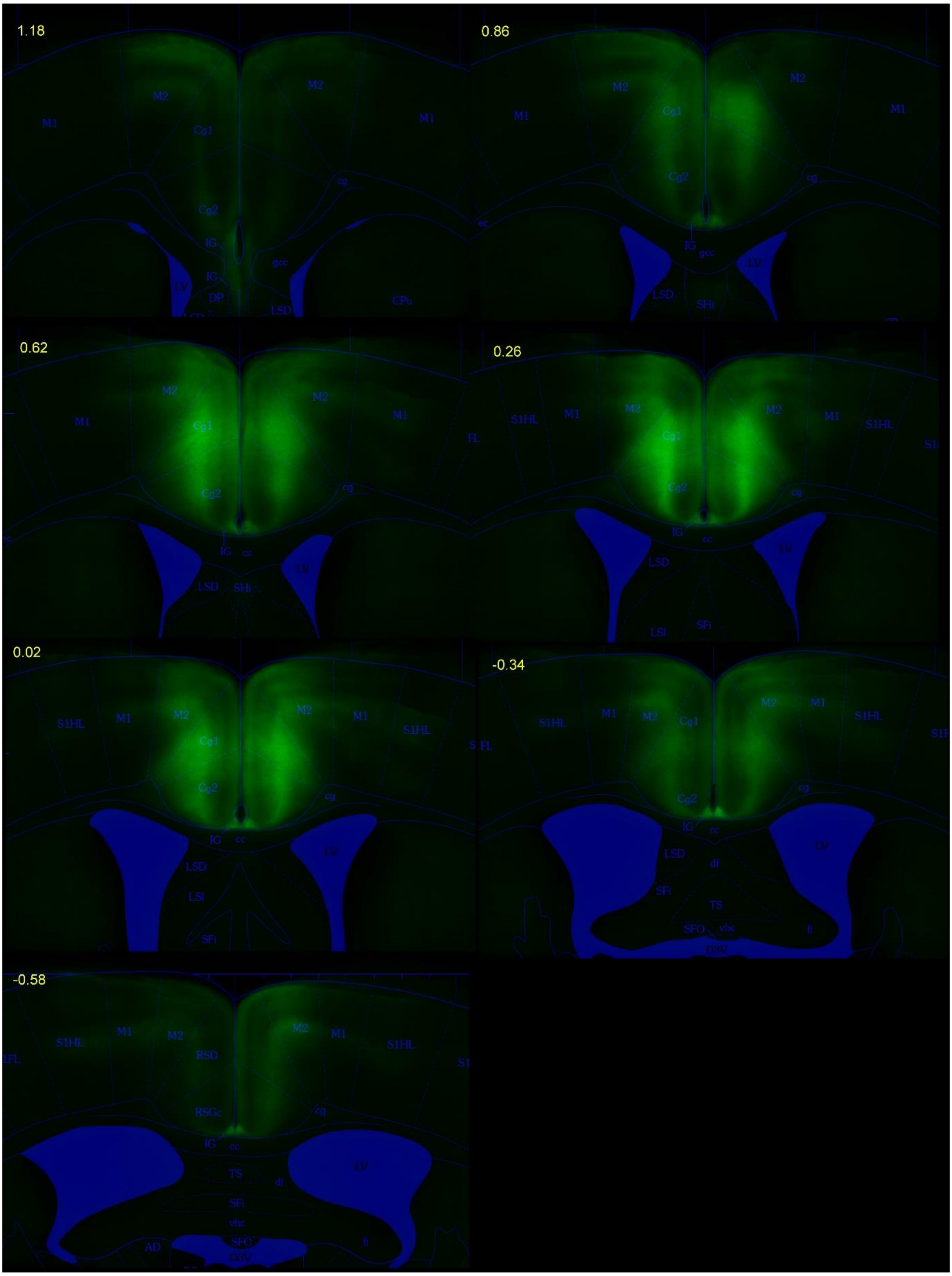

Figure S6. JAWS expression, related to figure 6. Average JAWS-GFP fluorescence for all JAWS-GFP animals included in the study aligned onto reference atlas (Paxinos and Franklin, 2007). Numbers indicate anterior-posterior position relative to bregma (mm).

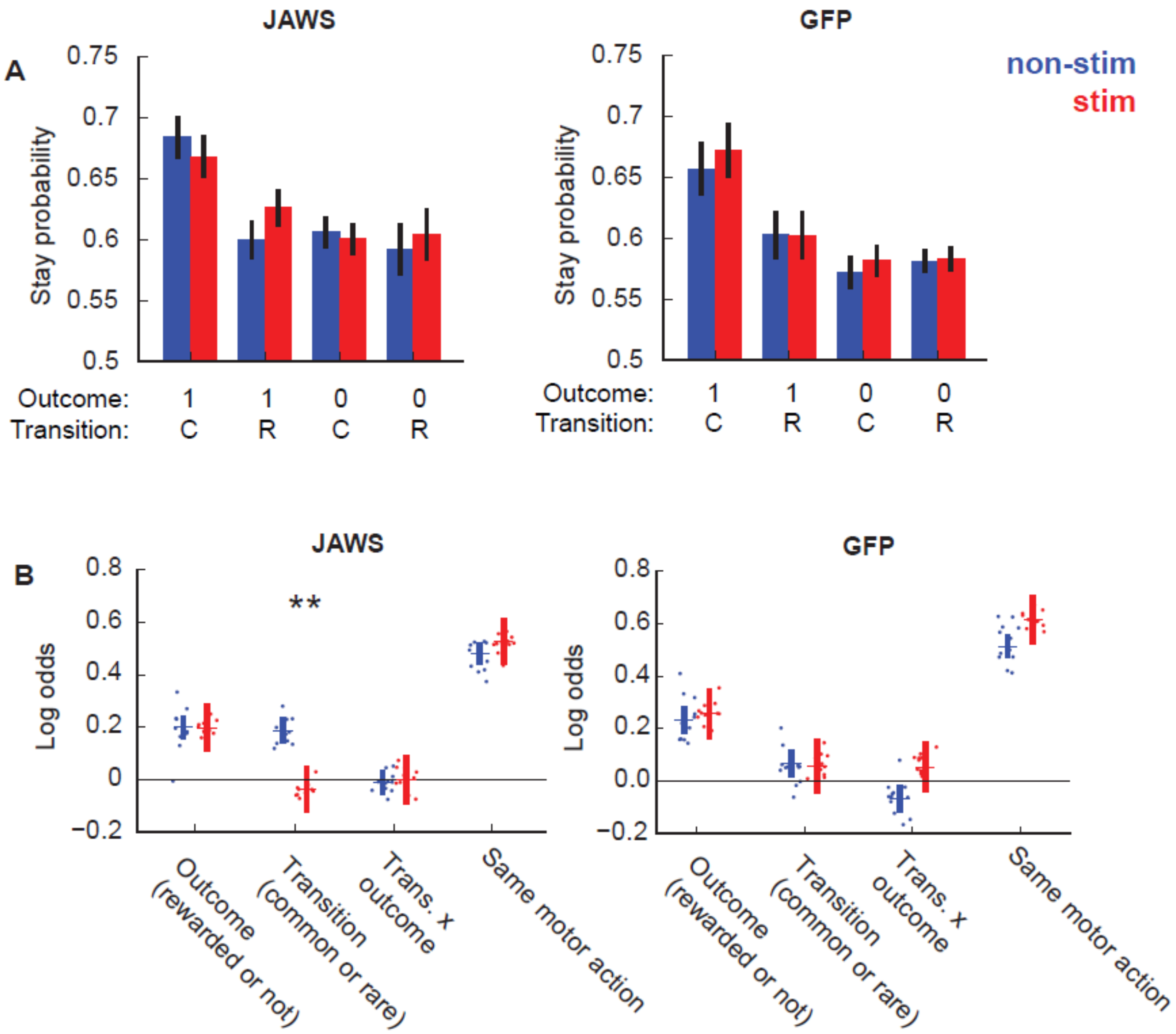

Figure S7. Optogenetic silencing of ACC in two-step task, related to figure 6. A) Stay probabilities analysis on stimulated (red) and non-stimulated (blue) trials in JAWS (top panel) and GFP (bottom panel). **B)** Evidence from RL model comparison for perseveration and model-free RL at the motor level raises a possible alternative interpretation of why ACC inhibition reduced the influence of common vs rare state transitions on choices. This is because the state transition determines which second-step state the subject ends up in, and hence the motor action required to repeat the choice on the next trial. To test whether motor-level factors can account for the ACC inhibition effect, we analysed the ACC inhibition data using a logistic regression analysis including an additional predictor *same motor action* which coded a tendency to repeat choices when this required the same motor action as the previous trial (e.g. left→top). Although *same motor action* significantly predicted repeating choice (P<0.0001, bootstrap test), ACC inhibition had no effect on the *same motor action* predictor (P=0.94 uncorrected), and the effect of ACC inhibition on the common/rare transition predictor remained significant (Bonferoni corrected P=0.0032, stim-by-group interaction P=0.032). This analysis, and the selective association between the strength of opto effect across subjects and the subjects use of model-based RL (Figure 6E), argue that the effect of ACC inhibition on sensitivity to action-state transitions is mediated by disrupted model-based RL and not motor level factors.

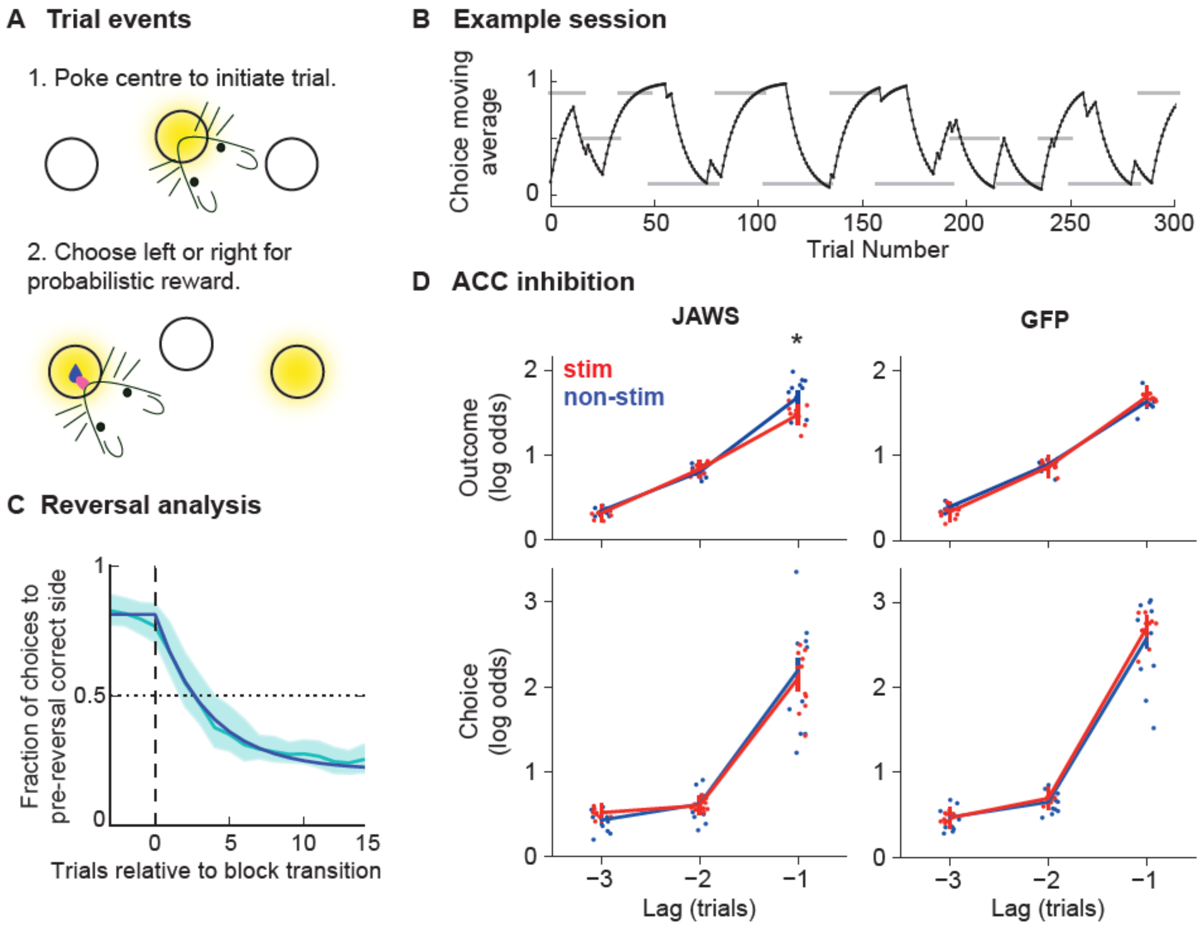

Figure S8. Optogenetic silencing of ACC in probabilistic reversal learning task, related to figure 6. A) Diagram of apparatus and trial events. **B)** Example session, black line shows exponential moving average (tau = 8 trials) of choices, grey bars indicate reward probability blocks with y position of bar indicating whether left or right side has high reward probability or a neutral block. **C)** Choice probability trajectories around reversal in reward probabilities: Pale blue line – average trajectory, dark blue line – exponential fit, shaded area – cross-subject standard deviation. **D)** Logistic regression analysis showing predictor loadings for stimulated (red) and non-stimulated (blue) trials, for the ACC JAWS (left panel) and GFP controls (right panel). Bars indicate 95% confidence intervals on the population mean, dots indicate maximum a posteriori (MAP) subject fits. * indicates significant difference ($P<0.05$, Bonferroni corrected for six predictors) between stimulated and non-stimulated trials.
