# Supplementary material for: The Anterior Cingulate Cortex Predicts Future States to Mediate Model-Based Action Selection
Source: Neuron. 2021 Jan 6;109(1):149–163.e7. doi: 10.1016/j.neuron.2020.10.013 (PMC7837117; doi:10.1016/j.neuron.2020.10.013)
Supplement: Document S2. Article plus Supplemental Information [file mmc2.pdf]

Neuron

Article

The Anterior Cingulate Cortex Predicts Future States to Mediate Model-Based Action Selection

Highlights

- A novel two-step task disambiguates model-based and model-free RL in mice
- ACC represents the task state space, and reward is contextualized by state
- ACC predicts future states given chosen actions and encodes state prediction surprise
- Inhibiting ACC prevents state transitions, but not rewards, from influencing choice

Authors

Thomas Akam, Ines Rodrigues-Vaz, Ivo Marcelo, ..., Rodrigo Freire Oliveira, Peter Dayan, Rui M. Costa

Correspondence

thomas.akam@psy.ox.ac.uk

In Brief

Akam et al. investigate mouse anterior cingulate cortex (ACC) in a sequential decision-making task, finding that ACC predicts future states given chosen actions and indicates when these predictions are violated. Transiently inhibiting ACC prevents mice from using observed state transitions to guide subsequent choices, impairing model-based reinforcement learning.

Akam et al., 2021, Neuron *109*, 149–163
January 6, 2021 © 2020 The Authors. Published by Elsevier Inc.
https://doi.org/10.1016/j.neuron.2020.10.013

CellPress

Neuron

CellPress
OPEN ACCESS

Article

The Anterior Cingulate Cortex Predicts Future States to Mediate Model-Based Action Selection

Thomas Akam,[1,2,9,*] Ines Rodrigues-Vaz,[1,3] Ivo Marcelo,[1,4] Xiangyu Zhang,[5] Michael Pereira,[1] Rodrigo Freire Oliveira,[1] Peter Dayan,[6,7,8] and Rui M. Costa[1,3]

[1]Champalimaud Neuroscience Program, Champalimaud Centre for the Unknown, Lisbon, Portugal
[2]Department of Experimental Psychology, Oxford University, Oxford, UK
[3]Department of Neuroscience and Neurology, Zuckerman Mind Brain Behavior Institute, Columbia University, New York, NY, USA
[4]Department of Psychiatry, Erasmus MC University Medical Center, 3015 GD Rotterdam, the Netherlands
[5]RIKEN-MIT Center for Neural Circuit Genetics at the Picower Institute for Learning and Memory, Department of Biology and Department of Brain and Cognitive Sciences, Massachusetts Institute of Technology, Cambridge, MA, USA
[6]Gatsby Computational Neuroscience Unit, University College London, London, UK
[7]Max Planck Institute for Biological Cybernetics, Tübingen, Germany
[8]University of Tübingen, Tübingen, Germany
[9]Lead Contact
*Correspondence: thomas.akam@psy.ox.ac.uk
https://doi.org/10.1016/j.neuron.2020.10.013

SUMMARY

Behavioral control is not unitary. It comprises parallel systems, model based and model free, that respectively generate flexible and habitual behaviors. Model-based decisions use predictions of the specific consequences of actions, but how these are implemented in the brain is poorly understood. We used calcium imaging and optogenetics in a sequential decision task for mice to show that the anterior cingulate cortex (ACC) predicts the state that actions will lead to, not simply whether they are good or bad, and monitors whether outcomes match these predictions. ACC represents the complete state space of the task, with reward signals that depend strongly on the state where reward is obtained but minimally on the preceding choice. Accordingly, ACC is necessary only for updating model-based strategies, not for basic reward-driven action reinforcement. These results reveal that ACC is a critical node in model-based control, with a specific role in predicting future states given chosen actions.

INTRODUCTION

Behavior is not a unitary phenomenon but rather is determined by partly parallel control systems that use different computational principles to evaluate choices (Balleine and Dickinson, 1998; Daw et al., 2005; Dolan and Dayan, 2013). A model-based controller learns to predict the specific consequences of actions (i.e., the states and rewards they immediately lead to) and evaluates their long-run utility by simulating behavioral trajectories. This confers behavioral flexibility, as the distant implications of new information can be evaluated using the model rather than learned through trial and error. However, the required simulations are computationally expensive and slow. Well-practiced actions in familiar environments are instead controlled by a habitual system, thought to involve model-free reinforcement learning (RL) (Sutton and Barto, 1998). This uses reward prediction errors to cache preferences between actions, allowing quick and computationally cheap decision making, at the cost of reduced behavioral flexibility.

Though model-based decision making is fundamental to flexible behavior, its implementation in the brain remains poorly understood. Mechanistically dissecting model-based control necessitates dissociating it from simpler model-free systems. This requires tasks in which each system recommends a different course of action. Historically, tasks that achieved this, such as outcome devaluation (Adams and Dickinson, 1981), were poorly suited to neurophysiology as they generated only a limited number of informative trials. More recently, sequential decision tasks for humans have been developed that disambiguate model-based and model-free control in a stable way over many trials. The most popular of these is the so-called two-step task (Daw et al., 2011), which has been used to probe mechanisms of model-based RL (Daw et al., 2011; Wunderlich et al., 2012; Smittenaar et al., 2013; Doll et al., 2015), arbitration between controllers (Keramati et al., 2011; Lee et al., 2014; Doll et al., 2016), and behavioral differences in psychiatric disorders (Sebold et al., 2014; Voon et al., 2015; Gillan et al., 2016). The original version of the task has also been adapted in work with rats and non-human primates (Miller et al., 2017; Dezfouli and Balleine, 2017; Hasz and Redish, 2018; Miranda et al., 2019; Groman et al., 2019).

This is an open access article under the CC BY license (http://creativecommons.org/licenses/by/4.0/).

Building on this work, we developed a novel two-step task for mice designed to dissociate state prediction from reward prediction in neural activity and model-based from model-free control in behavior. The task was additionally designed to prevent subjects from using alternative strategies that can otherwise complicate the interpretation of two-step task behavior in extensively trained animals (Akam et al., 2015).

We used this task to probe the involvement of the anterior cingulate cortex (ACC) in model-based decision making. The ACC is a critical contributor to reward guided decision making (Rushworth and Behrens, 2008; Heilbronner and Hayden, 2016) and is particularly associated with monitoring the outcomes of actions to update behavior (Hadland et al., 2003; Kennerley et al., 2006; Rudebeck et al., 2008). Diverse theoretical accounts have been offered for ACC function (Ebitz and Hayden, 2016), but an influential computational model proposes that many of the underlying observations can be accounted for by ACC generating precisely the type of specific action-outcome predictions required for model-based RL (Alexander and Brown, 2011). However, despite evidence suggestive of ACC's involvement in model-based reinforcement (Daw et al., 2011; Cai and Padoa-Schioppa, 2012; Karlsson et al., 2012; O'Reilly et al., 2013; Doll et al., 2015; Huang et al., 2020), tasks designed to dissociate model-based and model-free control have not to our knowledge been combined with single-unit recordings or causal manipulations in ACC.

Combining a sequential decision task with calcium imaging and optogenetics, our data demonstrate a rich set of task representations in ACC, including action-state predictions and surprise signals, and a causal role in using observed action-state transitions to guide subsequent choices. These results reveal that ACC is a critical component of the model-based controller and uncover a neural basis for predicting future states given chosen actions.

RESULTS

A Novel Two-Step Task with Transition Probability Reversals

As in the original two-step task (Daw et al., 2011), our task consisted of a choice between two "first-step" actions that led probabilistically to one of two "second-step" states in which reward could be obtained. Each first-step action commonly led to one second-step state and rarely to the other. However, whereas in the original task these action-state transition probabilities were constant, we introduced occasional reversals in the transition probabilities (i.e., transitions that were previously common became rare and vice versa).

Transition probability reversals have two desirable consequences. First, if both reward and action-state transition probabilities change independently over time, it is possible to dissociate state prediction and reward prediction in neural activity. Second, reversals in the transition probabilities prevent subjects from using habit-like strategies consisting of mappings from the second-step state in which rewards have recently been obtained to specific actions at the first step. This can in principle generate behavior that looks very similar to model-based control, despite not using forward planning (Akam et al., 2015). Transition probability reversals break the long-run predictive relationship between where rewards are obtained and which first-step action is correct, preventing these strategies while still permitting model-based RL. We directly compared versions of the task with fixed and changing action-state transition probabilities (Figure S1) and found that subject's behavior was radically different in each, suggesting that they recruit different strategies.

To simplify the task for mice, we used a single action available in each second-step state rather than the choice between two actions in the original task. We also increased the contrast between good and bad options, as in the original task the stochasticity of state transitions and reward probabilities causes both model-based and model-free control to obtain rewards at a rate negligibly different from random choice at the first step (Akam et al., 2015; Kool et al., 2016). To promote task engagement, we therefore used a block-based reward probability distribution rather than the random walks used in the original and increased the probability of common relative to rare state transitions.

We physically implemented the task using a set of four nose-poke ports: top and bottom ports in the center, flanked by left and right ports (Figure 1A). Each trial started with the central ports lighting up, requiring a choice between top and bottom ports. The choice of a central port led probabilistically to a "left-active" or "right-active" state, in which respectively the left or right port was illuminated. The subject then poked the illuminated left or right port to gain a probabilistic water reward (Figures 1A and 1B). Pokes to non-illuminated ports were ignored, so at the first step only pokes to the top or bottom ports, and at the second step only pokes to the illuminated side port, affected the task. A 1 second inter-trial interval started when the subject exited the side port. Subjects rarely poked either side port at the time of first-step choice, or the inactive side port at the second step (Figure S2), indicating that they understood the trial structure.

Each block was defined by the state of both the reward and transition probabilities (Figure 1C). There were three possible states of the reward probabilities for the left/right ports: respectively good/bad, neutral/neutral, and bad/good, where good/neutral/bad reward probabilities were 0.8/0.4/0.2. There were two possible states of the transition probabilities: top → left/bottom → right and top → right/bottom → left (Figure 1C), where, for example, top → right indicates that the top port commonly (0.8 of trials) led to the right port and rarely (0.2 of trials) to the left port. At block transitions, the reward and/or transition probabilities changed (see STAR Methods). Reversals in which first-step action (top or bottom) had higher reward probability could therefore occur because of reversals in either the reward or transition probabilities. Block transitions were triggered on the basis of a behavioral criterion (see STAR Methods) that resulted in block lengths of 63.6 ± 31.7 (mean ± SD) trials.

Subjects learned the task in 3 weeks with minimal shaping and performed an average of 576 ± 174 (mean ± SD) trials per day thereafter (Table 1). Our behavioral dataset used data from day 22 of training onward (n = 17 mice, 400 sessions, 230,237 trials). Subjects tracked which first-step action had higher reward probability (Figures 1D and 1E), choosing the correct option at the end of non-neutral blocks with probability 0.68 ± 0.03 (mean ± SD). Choice probabilities adapted faster following reversals in the

CellPress
OPEN ACCESS

Table 1. Two-Step Task Parameter Changes over Training

Session Number	Reward Size (µl)	Transition Probabilities (Common/Rare)	Reward Probabilities (Good/Bad Side)
1	10	0.9/0.1	first 40 trials all rewarded, subsequently 0.9/0.1
2–4	10	0.9/0.1	0.9/0.1
5–6	6.5	0.9/0.1	0.9/0.1
7–8	4	0.9/0.1	0.9/0.1
9–12	4	0.8/0.2	0.9/0.1
≥13	4	0.8/0.2	0.8/0.2

action-state transition probabilities (exponential fit tau = 17.6 trials), compared with reversals in the reward probabilities (tau = 22.7 trials, p = 0.009, bootstrap test; Figure 1E).

Reaction times to enter the second-step port were faster following common than rare transitions ($p = 2.8 \times 10^{-8}$, paired t test) (Figure 1F). However, in our task (unlike the original), the motor action associated with a given second-step state is fixed, and hence second-step reaction time differences may reflect preparatory activity at the motor level and so may not provide strong evidence about subjects' decision strategy.

The Novel Task Disambiguates Model-Based and Model-Free Control in Mice

To assess ACC's involvement in model-based and model-free control, we require that the task recruit both systems and disambiguate the contribution of each to behavior. In the original two-step task, the contribution of each systems can be assessed by examining the so-called stay probabilities of repeating the first-step choice as a function of subsequent trial events. Model-based control causes the interaction of state transition (common or rare) and outcome (rewarded or not) to determine stay probabilities (Daw et al., 2011). This is because rewards following common transitions promote repeating the same choice on the next trial, but rewards following rare transitions increase the value of the state commonly accessed via the not-chosen first-step action and hence promote switching. Model-free control by contrast causes the outcome, but not transition, to determine stay probabilities, because rewards directly reinforce actions that precede them irrespective of the transition that occurred.

We expect this picture to be somewhat different in the present task. In the original two-step task, it is assumed that subjects do not update their estimates of the transition probabilities in light of experienced state transitions, because the transition probabilities are fixed, and subjects are explicitly told this. In our task the transition probabilities change over time, so a model-based controller must update transition probability estimates on the basis of experience. We have previously shown that when such model learning is included, the influence of transition-outcome interaction on stay probability is reduced, but common transitions themselves become reinforcing (Akam et al., 2015). This is because a model-based agent chooses the first-step action it believes will reach the better of the two second-step states. Common transitions confirm the agent in its belief that the chosen action reaches the desired state, while rare transitions make it appear more likely that the not-chosen action reaches the better state.

Table 2. RL and Logistic Regression Model Variables and Parameters

Variables and Parameters	Description
Logistic Regression Model Predictors	
Bias: top/bottom	choose top-poke
Bias: clockwise/counterclockwise	choose top if previous trial ended at left poke, bottom if at right
Choice	repeat choice
Correct	repeat correct choice
Outcome	repeat rewarded choice
Transition	repeat choice followed by common transition
Transition-outcome interaction	repeat choice followed by rewarded common and non-rewarded rare transitions
RL Model Variables	
r	reward (0 or 1)
c	choice taken at first step (top or bottom poke)
c'	choice not taken at first step (top or bottom poke)
s	second-step state (left-active or right-active)
s'	state not reached at second step (left-active or right-active)
$Q_{mf}(c)$	model-free action value for choice c
$Q_{mo}(c,s_{t-1})$	motor-level model-free action value for choice c following second-step state s_{t-1}
$Q_{mb}(c)$	model-based value of choice c
$V(s)$	value of state s
$P(s\|c)$	estimated transition probability of reaching state s after choice c
$\bar{c}$	choice history
$\overline{m}(s_{t-1})$	motor action history (i.e., choice history following second-step state s_{t-1})
RL Model Parameters	
α_Q	value learning rate
f_Q	value forgetting rate
λ	eligibility trace parameter
α_T	transition learning rate
f_T	transition forgetting rate
α_c	learning rate for choice perseveration
α_m	learning rate for motor-level perseveration
G_{mf}	model-free action value weight
G_{mo}	motor-level model-free action value weight
G_{mb}	model-based action value weight
B_c	choice bias (top/bottom)
B_r	rotational bias (clockwise/counterclockwise)
P_c	choice perseveration strength
P_m	motor-level perseveration strength

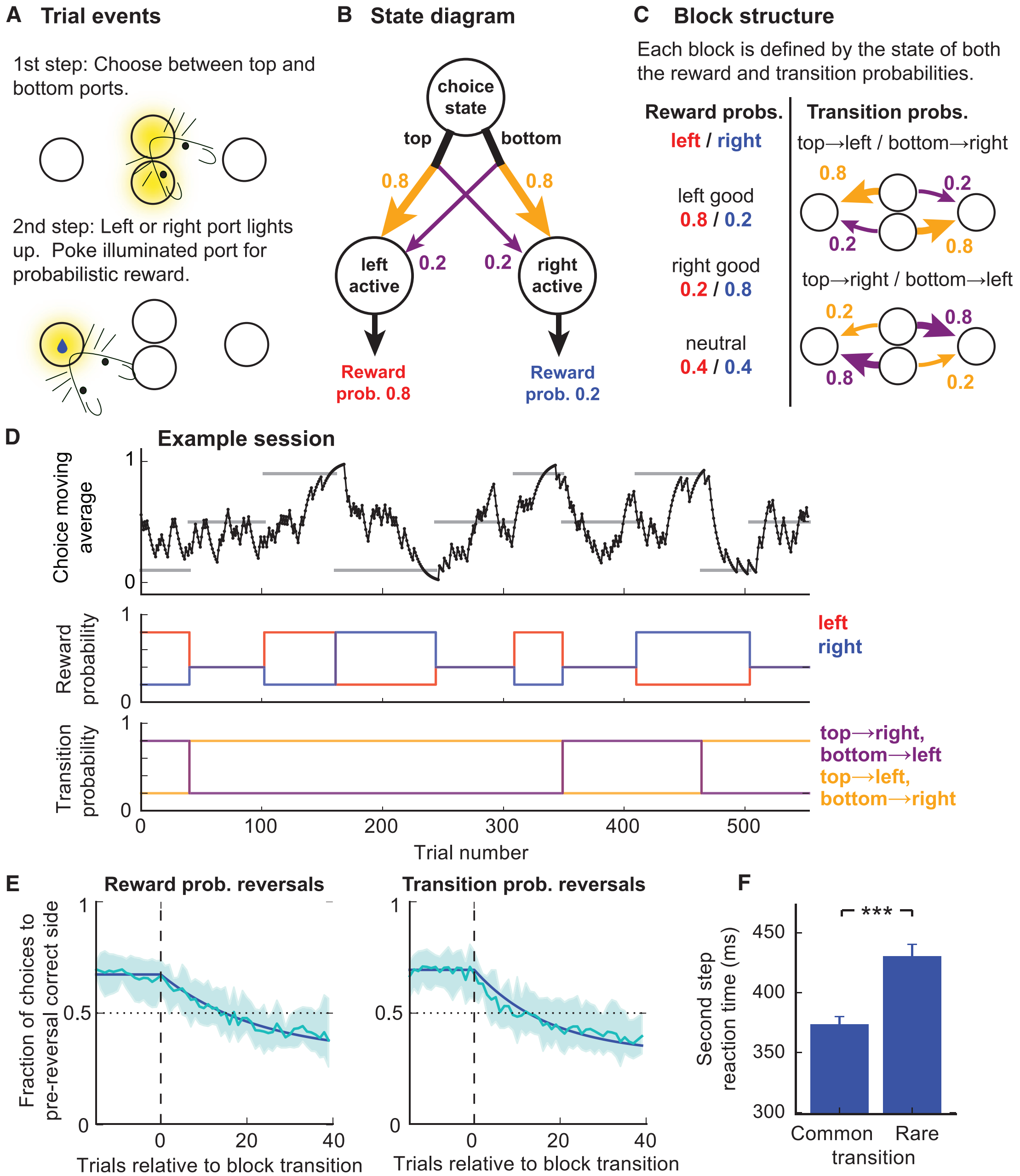

Figure 1. Two-Step Task with Transition Probability Reversals

(A) Diagram of apparatus and trial events.

(B) State diagram of task. Reward and transition probabilities are indicated for one of the six possible block types.

(C) Block structure; left side shows the three possible states of the reward probabilities, right side shows the two possible states of the transition probabilities.

(D) Example session. Top panel: exponential moving average (tau = 8 trials) of choices. Horizontal gray bars show blocks, with correct choice (top, bottom, or neutral) indicated by y position of bars. Middle panel: reward probabilities in left-active (red) and right-active (blue) states. Bottom panel: transition probabilities linking first-step actions (top, bottom pokes) to second-step states (left/right active).

(E) Choice probability trajectories around reversals. Pale blue line, average trajectory; dark blue line, exponential fit; shaded area, cross-subject SD. Left panel: reversals in reward probability; right panel: reversals in transition probabilities.

(F) Second step reaction times following common and rare transitions (i.e., the time between the first-step choice and side poke entry). ***$p < 0.001$ Error bars show cross-subject SEM.

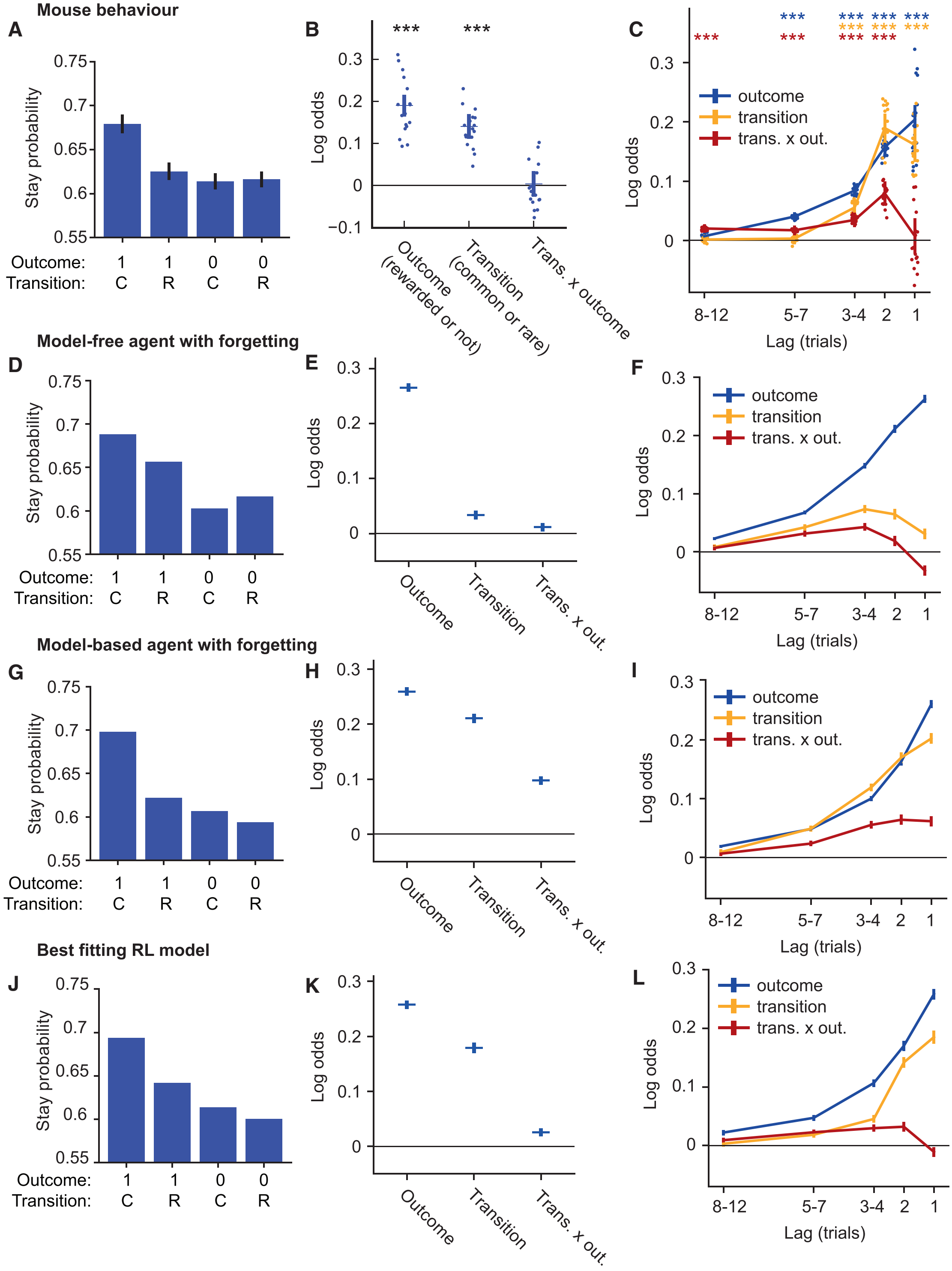

Figure 2. Stay Probability and Logistic Regression Analyses

(A–C) Mouse behavior. (A) Stay probability analysis showing the fraction of trials the subject repeated the same choice following each combination of trial outcome (rewarded [1] or not [0]) and transition (common [C] or rare [R]). Error bars show cross-subject SEM. (B) Logistic regression model fit predicting choice as a function of the previous trial's events. Predictor loadings plotted are outcome (repeat choices following rewards), transition (repeat choices following common transitions), and transition-outcome interaction (repeat choices following rewarded common transition trials and non-rewarded rare transition trials). Error bars indicate 95% confidence intervals on the population mean, dots indicate maximum a posteriori (MAP) subject fits. (C) Lagged logistic regression model predicting choice as a function of events over the previous 12 trials. Predictors are as in (B).

(legend continued on next page)

We quantified how transition, outcome, and their interaction predicted stay probability in the present task (Figure 2A) using a logistic regression analysis (Figure 2B), with additional predictors to capture choice biases and correct for cross-trial correlations which can otherwise can give a misleading picture of how trial events influence subsequent choice (Akam et al., 2015; Table 2). Positive loading on the outcome predictor indicated that reward was reinforcing (i.e., predicted staying) ($p < 0.001$, bootstrap test). Positive loading on the transition predictor indicated that common transitions were also reinforcing ($p < 0.001$), as expected for model-based control with transition probability learning. Loading on the transition-outcome interaction predictor was not significantly different from zero ($p = 0.79$). To understand the implications of this, we simulated the behavior of a model-based and a model-free RL agent, with the parameters of both fit to the behavioral data, and ran the logistic regression analysis on data simulated from both models (Figures 2D–2I). The RL agents used in these simulations included forgetting about actions not taken and states not visited, as RL model comparison indicated this greatly improved fits to mouse behavior (see below). Data simulated from a model-free agent showed a large loading on the outcome predictor (i.e., rewards were reinforcing) but little loading on the transition predictor or transition-outcome interaction predictors (Figure 2E). In contrast, data simulated from the model-based agent showed a large loading on both outcome and transition predictors (i.e., both rewards and common transitions were reinforcing) (Figure 2H) and a smaller loading on the interaction predictor. Therefore, in our data the transition predictor loaded closer to the model-based strategy, and the interaction predictor loaded closer to the model-free strategy.

The above analysis considers only the influence of the most recent trial's events on choice. However, the slow time course of adaptation to reversals (Figure 1E) indicates that choices must be influenced by a longer trial history. To better understand these long-lasting effects, we used a lagged regression analysis assessing how the current choice was influenced by past transitions, outcomes, and their interaction (Figure 2C). Predictors were coded such that a positive loading on, for example, the outcome predictor at lag x indicates that reward on trial t increased the probability of repeating the trial t choice at trial $t + x$. Past outcomes significantly influenced current choice up to lags of seven trials, with a smoothly decreasing influence at larger lags. Past state transitions influenced the current choice up to lags of four trials with, unexpectedly, a somewhat larger influence at lag 2 compared with lag 1. Also unexpectedly, although the transition-outcome interaction on the previous trial did not significantly influence the current choice, the interaction at lag 2 and earlier did, with the strongest effect at lag 2.

To understand how these patterns relate to RL strategy, we analyzed the behavior of model-based and model-free agents using the lagged regression (Figures 2F and 2I). Subjects behavior did not closely resemble either pure strategy, nor did it appear to be a simple mixture, suggesting the presence of additional features. To assess how behavior diverged from these models, we performed an in-depth model comparison, detailed in Figure S3. The best fitting model used a mixture of model-based and model-free control but also incorporated additional features not typically used to model two-step task behavior: forgetting about values and state transitions for not-chosen actions, perseveration effects spanning multiple trials, and representation of actions both at the level of the choice they represent (e.g., top port) and the motor action they require (e.g., left port → top port). Taken together, the additional features substantially improved fit quality (Δ integrated Bayes information criterion [iBIC] = 11,018), and data simulated from the best fitting RL model better matched mouse behavior (Figures 2J–2L). These data indicate that the novel task recruits both model-based and model-free RL mechanisms, providing a tool for mechanistic investigation into mechanism of flexible and automatic behavior in the mouse.

ACC Activity Represents the Task State-Action Space, and Reward Is Contextualized by State

We expressed GCaMP6f in ACC pyramidal neurons under the CaMKII promotor and imaged calcium activity through a gradient refractive index (GRIN) lens using a miniature fluorescence microscope (n = 4 mice, 21 sessions, 2,385 neurons, 3,732 trials) (Ghosh et al., 2011). Constrained non-negative matrix factorization for endoscope data (CNMF-E) (Zhou et al., 2018) was used to extract activity traces for individual neuron from the microscope video (Figure 3B). All subsequent analyses used the deconvolved activity inferred by CNMF-E. Activity was sparse, with an average event rate of 0.12 Hz across the recorded population (Figure 3C). We aligned activity across trials by time-warping the interval between the first-step choice and second-step port entry (labeled "outcome" in figures, as this is when outcome information becomes available) to match the median interval (Figure S4). Activity prior to choice and following outcome was not time warped.

Different populations of neurons participated at different time points across the trial (Figure 3D; Figure S5). Many ACC neurons ramped up activity over the 1,000 ms preceding the first step-choice, peaking at choice time and being largely silent following trial outcome. Other neurons were active in the period between choice and outcome, and yet others were active immediately following trial outcome. Individual neurons showed strong tuning to trial events, particularly the choice and second-step state, and to conjunctions of choice and second step or second step and outcome (Figure S5).

To characterize how the population represented events in the present trial, we used a linear regression predicting the activity of each neuron at each time point as a function of the choice (top or bottom), second-step state (left or right), and outcome (rewarded or not) that occurred on the trial, as well as the interactions between these events. This and later analyses included

(D–F) As (A)–(C) but for data simulated from a model-free RL agent with forgetting and multi-trial perseveration.
(G–I) As (A)–(C) but for data simulated from a model-based RL agent with forgetting and multi-trial perseveration.
(J–L) As (A)–(C) but for data simulated from the best fitting RL model found by model comparison.
Parameters for all RL model simulations were obtained by fits of the RL models to the mouse behavioral data.

CellPress
OPEN ACCESS

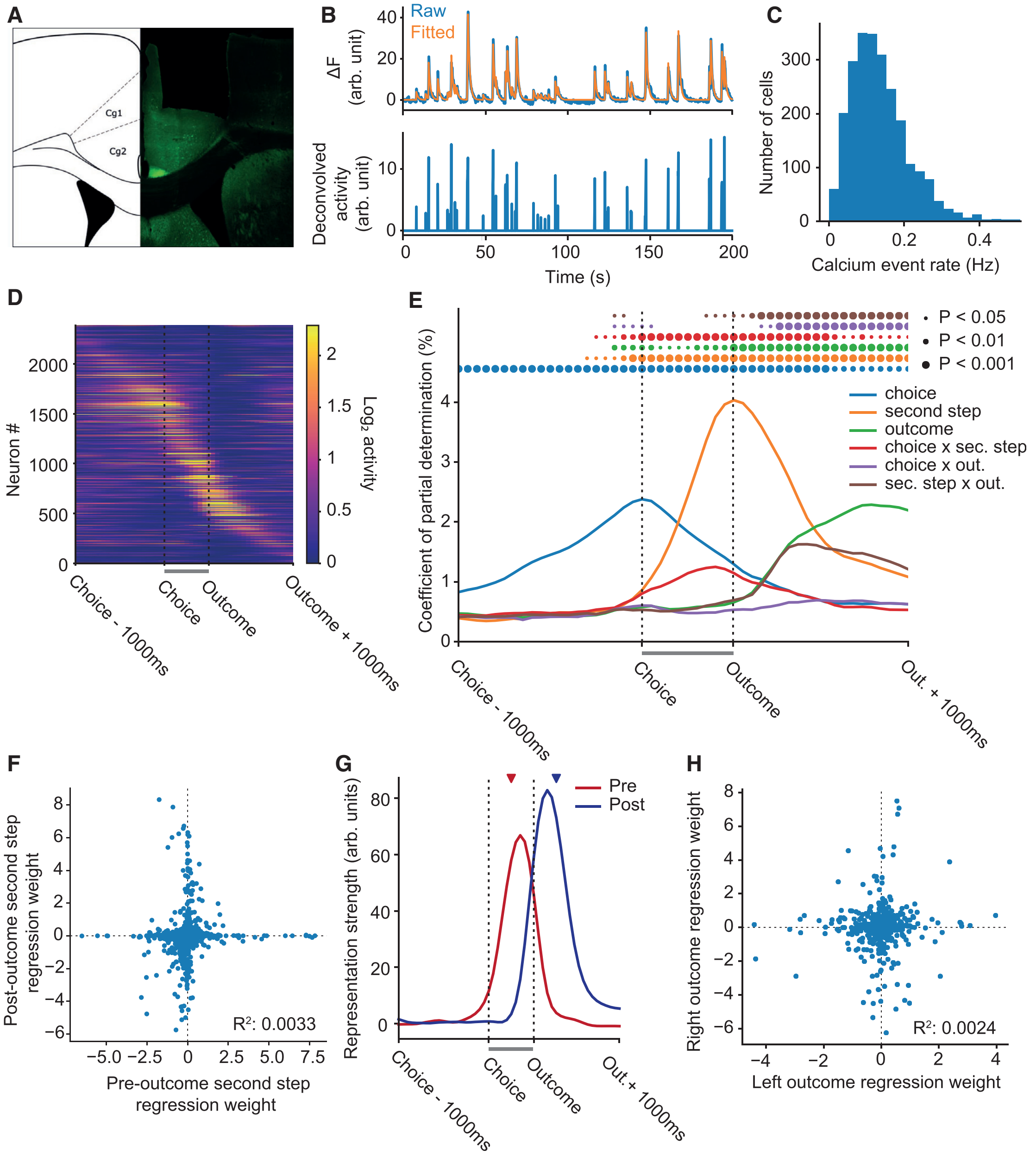

Figure 3. Two-Step ACC Calcium Imaging

(A) Example GRIN lens placement in ACC.

(B) Fluorescence signal from a neuronal region of interest (ROI) identified by CNMF-E (top panel, blue) and fitted trace (orange) due to the inferred deconvolved neuronal activity (bottom panel).

(C) Histogram showing the distribution of average event rates across the population of recorded neurons. Events were defined as any video frame on which the inferred activity was non-zero.

(D) Average trial aligned activity for all recorded neurons, sorted by the time of peak activity. No normalization was applied to the activity. The gray bars under (D), (E), and (G) between choice and outcome indicate the time period that was warped to align trials of different duration.

(E) Regression analysis predicting activity on each trial from a set of predictors coding the choice (top or bottom), second step (left or right), outcome (rewarded or not) that occurred in each trial, and their interactions. Lines show the population coefficient of partial determination (CPD) as a function of time relative to trial events. Circles indicate where CPD is significantly higher than expected by chance, assessed by permutation test with Benjamini-Hochberg correction for comparison at multiple time points.

(F) Representation of the second-step state before and after the trial outcome. Points show second-step predictor loadings for individual neurons at a time point halfway between choice and outcome (x axis) and a time point 250 ms after trial outcome (y axis).

(G) Time course of pre- and post-outcome representations of second-step state, obtained by projecting the second step predictor loadings at each time point onto the pre- and post-outcome second-step representations. The red and blue triangles indicate the time points used to define the projection vectors.

(legend continued on next page)

only sessions for which we had sufficient coverage of all trial types (n = 3 mice, 11 sessions, 1,314 neurons, 2,671 trials), as in some imaging sessions with few blocks and trials there was no coverage of trial types that occur infrequently in those blocks. We evaluated the population coefficient of partial determination (i.e., the fraction of variance across the population uniquely explained by each predictor) as a function of time relative to trial events (Figure 3E). Representation of choice ramped up smoothly over the second preceding the choice, then decayed smoothly until approximately 500 ms after trial outcome. Representation of second-step state increased rapidly following the choice, peaked at second-step port entry, then decayed over the second following the outcome and was the strongest represented trial event.

As partially distinct populations of neurons were active before and after trial outcome (Figures 3D and S5), we asked whether the population representation of second-step state was different at these two time points. We plotted the second-step state regression weights for each neuron at a time point mid-way between choice and outcome (which we term the pre-outcome representation of second-step state) against the weights 250 ms after outcome (the post-outcome representation) (Figure 3F). These pre- and post-outcome representations were uncorrelated (R^2 = 0.0033), indicating that although second-step state was strongly represented at both times, the representations were orthogonal and involved different populations of neurons. To evaluate the time course of these two representations, we projected the second-step state regression weights at each time point across the trial onto the two representations (Figure 3G), using cross-validation to give an unbiased time course estimates. The pre-outcome representation of second-step state peaked shortly before second-step port entry and decayed rapidly afterward, while the post-outcome representation peaked shortly after trial outcome and persisted for ~500 ms.

Representation of the trial outcome ramped up following receipt of outcome information (Figure 3E), accompanied by an initially equally strong representation of the interaction between trial outcome and second-step state. This interaction indicates that the representation of trial outcome depended strongly on the state in which the outcome was received, and individual neurons which differentiated between reward and non-reward tended to do so only in one of the two second-step states (Figure S5). To assess this in more detail, we ran a version of the regression analysis with separate predictors for outcomes received at the left and right ports, and plotted the left and right outcome regression weights 250 ms after outcome against each other (Figure 3H). Representations of trial outcome obtained at the left and right ports were orthogonal (R^2 = 0.0024), indicating that although ACC carried information about reward, reward representations were specific to the state where the reward was received.

The evolving representation of trial events can be visualized by projecting the average neuronal activity for each trial type (defined by choice, second-step state, and outcome) into the low dimensional space that captures the greatest variance between different trial types (see STAR Methods) (Figure 4). The first three principal components (PCs) of this space were dominated by representation of choice and second-step state (Figures 4A and 4B), with different trial outcomes being most strongly differentiated in PC4 and PC5 (Figure 4C). Prior to the choice, trajectories diverged along an axis capturing choice selectivity (PC2). Following the choice, trajectories for different second-step states diverged first along one axis (PC3), then along a second axis (PC1), confirming that two orthogonal representations of second-step state occur in a sequence spanning the time period from choice through trial outcome.

To quantify how accurately ACC activity differentiated between task states, we decoded which of ten different locations in the task's state-action space neuronal activity came from, using a multinomial logistic regression. Locations were defined by time point in the trial (pre-choice, post-choice, and post-outcome) and the trial's choice, second step, and outcome (Figure 4D). The analysis combined activity from 1,053 neurons from the nine sessions in which each location was visited at least ten times, yielding a cross-validated decoding accuracy of 95% (Figure 4E), where chance level is 10%. These data show that ACC activity represents the full set of trial events that constitute the state-action space of the task.

ACC Represents Model-Based Decision Variables

Model-based RL uses predictions of the specific consequences of action (i.e., the states that actions lead to) to compute their values. Therefore if ACC implements model-based computations, we expect to see predictions of future state given chosen action and surprise signals if these predictions are violated, both of which require knowledge of the current configuration of the transition probabilities linking first-step actions to second-step states.

We therefore asked how ACC activity was affected by the changing transition probabilities mapping the first-step actions to second-step states and reward probabilities in the second-step states. Because of the limited number of blocks that subjects performed in imaging sessions, we performed separate regression analyses for sessions for which we have sufficient coverage of the different states of the transition probabilities (Figure 5A; n = 3 mice, 5 sessions, 589 neurons, 1,252 trials) and reward probabilities (Figure 5B; n = 3 mice, 10 sessions, 1,152 neurons, 2,426 trials). These analyses predicted neuronal activity as a function of events in the current trial, the state of the transition or reward probabilities respectively, and their interactions. Though each analysis used only a subset of imaging sessions, the representation of current trial events (Figures 5A and 5B, top panels) was in both cases very similar to that for the full dataset (Figure 3E). As both the transition and reward probabilities determine which first-step action is correct, effects common to these two analyses could in principle be mediated by

(H) Representation of trial outcomes (reward or not) obtained at the left and right poke. Points show predictor loadings for individual neurons 250 ms after trial outcome in a regression analysis in which outcomes at the left and right poke were coded by separate predictors. The regression analysis was identical to that shown in (E) except that the outcome and second-step x outcome predictors were replaced by left outcome and right outcome predictors, which coded reward/non-reward in trials that reached the left or right second-step state, respectively.

CellPress
OPEN ACCESS

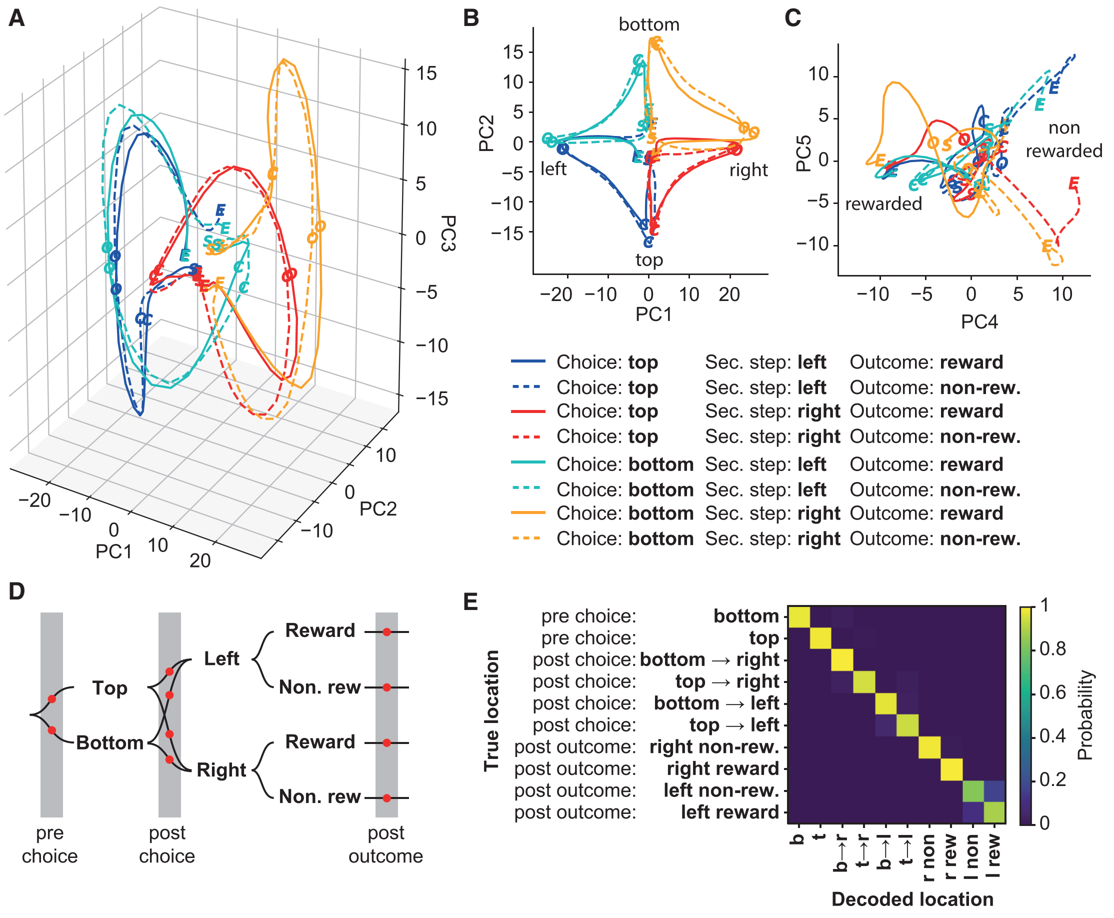

Figure 4. ACC Represents the Full State-Action Space

(A–C) Projection of the average population activity for different trial types into the low-dimensional space that captures the most variance between trial types. Trial types were defined by the eight combinations of choice, second step, and trial outcome. Letters on the trajectories indicate the trajectory start (S; 1,000 ms before choice), the choice (C), outcome (O), and trajectory end (E; 1,000 ms after outcome). (A) Three-dimensional plot showing projections onto first three principal components. (B) Projection onto PC1 and PC2, which represent second-step and choice, respectively. (C) Projection onto PC4 and PC5, which differentiate trial outcomes.

(D and E) Decoding analysis assessing how accurately ACC population activity differentiates between different locations in task's state-action space. (D) Diagram showing the ten different locations (red dots) in the tasks state-action space used in the decoding analysis. (E) Confusion matrix showing the cross-validated probability of decoding each location given the actual location the activity was from.

changes in first-step action values rather than the reward or transition probabilities themselves, but effects that are specific to one or other analysis cannot.

Representation of the current state of the transition probabilities (Figure 5A, cyan), but not reward probabilities (Figure 5B, cyan), ramped up prior to choice and was sustained through trial outcome, though was significant only in the pre-choice period. Representation of the predicted second-step state given the current choice (the interaction of the choice on the current trial with the state of the transition probabilities) also ramped up prior to choice (Figure 5A, gray), peaking around choice time. Though ACC represented the interaction of choice with the reward probabilities (Figure 5B, gray), the time course was different, with weak representation prior to choice and a peak shortly before trial outcome.

Once the second-step state was revealed, ACC represented whether the transition was common or rare (i.e., the interaction of the transition on the current trial with the state of the transition probabilities) (Figure 5A, magenta). There was no representation of the equivalent interaction of the transition on the current trial with the state of the reward probabilities (Figure 5B, magenta). Finally, ACC represented the interaction of the second-step state reached on the current trial with both the transition and reward probabilities, with both representations ramping up after the second-step state was revealed and persisting till after trial outcome (Figures 5A and 5B, yellow). The interaction of second-step state with the transition probabilities corresponds to the action that commonly leads to the second-step state reached, potentially providing a substrate for model-based credit assignment. The interaction of second-step state with the reward probabilities corresponds to the predicted trial outcome (rewarded or not).

These data show that ACC represents a set of decision variables required for model-based RL, including the current

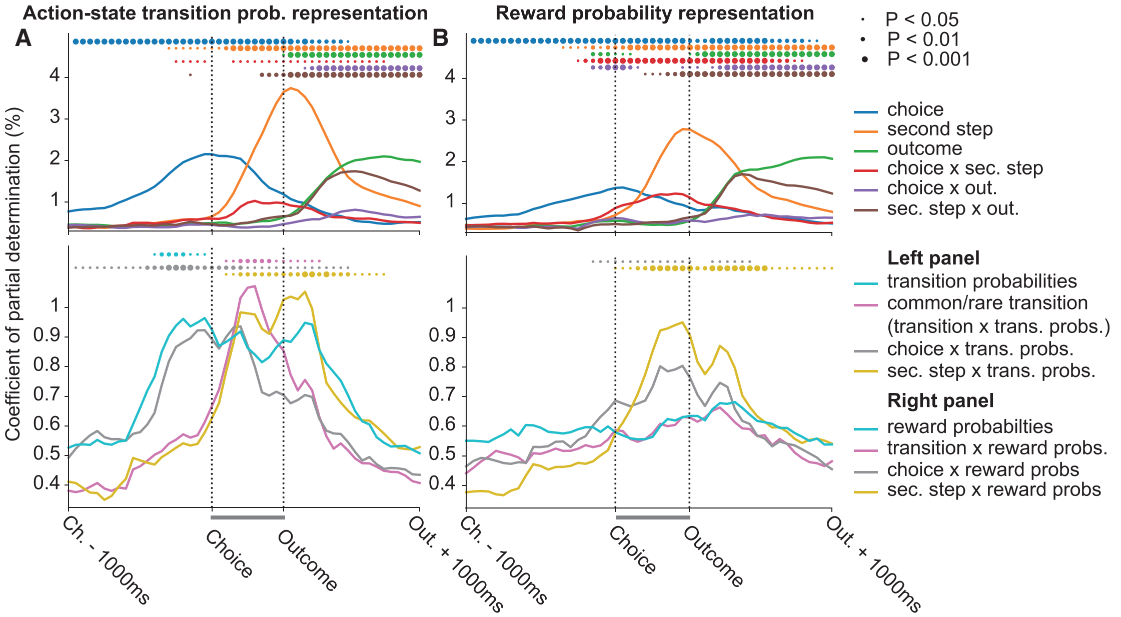

Figure 5. ACC Represents Model-Based Decision Variables

(A) Regression analysis predicting neuronal activity as a function of events in the current trial (top panel) and their interaction with the transition probabilities (trans. probs.) mapping the first-step choice to second-step (sec. step) states (bottom panel) for a subset of sessions with sufficient coverage of both states of the transition probabilities. Predictors plotted in top panels are as in Figure 3E. Predictors plotted in the bottom panel are transition probabilities (which of the two possible states the transition probabilities are in; see Figure 1C), common/rare transition (whether the transition on the current trial was common or rare, i.e., the interaction of the transition on the current trial [e.g., top → right] with the state of the transition probabilities), choice × trans. probs. (the choice in the current trial interacted with the state of the transition probabilities, i.e., the predicted second-step state given the current choice), and sec. step × trans. probs. (the second-step state reached on the current trial interacted with the state of the transition probabilities, i.e., the action which commonly leads to the second-step state reached). Predictors shown in top and bottom panels of (A) were run as a single regression but plotted on separate axes for clarity. The gray bars between choice and outcome indicate the time period that was warped to align trials of different length. Circles indicate where CPD is significantly higher than expected by chance, assessed by permutation test with Benjamini-Hochberg correction for comparison at multiple time points.

(B) Regression analysis predicting neuronal activity as a function of events on the current trial (top panel) and their interaction with the reward probabilities in the second-step states (bottom panel) for a subset of sessions with sufficient coverage of different states of the reward probabilities. Predictors plotted in the bottom panel are reward probabilities (which of the three possible states the transition probabilities are in; see Figure 1C), transition × reward probs. (interaction of the transition in the current trial with the state of the reward probabilities), choice × reward probs. (the choice in the current trial interacted with the state of the reward probabilities), and sec. step × trans. probs. (the second-step state reached in the current trial interacted with the state of the rewarded probabilities, i.e., the expected outcome [rewarded or not]. Predictors shown in top and bottom panels of (B) were run as a single regression but plotted on separate axes for clarity.

action-state transition structure, the predicted state given chosen action, and whether the observed state transition was expected or surprising.

Single-Trial Optogenetic Inhibition of ACC Impairs Model-Based RL

To test whether ACC activity is necessary for model-based control, we silenced ACC neurons on individual trials using JAWS (Chuong et al., 2014). An adeno-associated virus (AAV) viral vector expressing JAWS-GFP under the CaMKII promotor was injected bilaterally into ACC of experimental animals (n = 11 mice, 192 sessions, 77,350 trials) (Figure S6), while GFP was expressed in control animals (n = 12 mice, 197 sessions, 71,071 trials). A red light-emitting diode (LED) was chronically implanted above the cortical surface (Figure 6A). Electrophysiology confirmed that red light (50 mW, 630 nM) from the implanted LED robustly inhibited ACC neurons (Figure 6B; Kruskal-Wallis $p < 0.05$ for 67 of 249 recorded cells). ACC neurons were inhibited on a randomly selected 1 of 6 trials, with a minimum of 2 non-stimulated trials between each stimulation. Light was delivered from the time when the subject entered the side port and received the trial outcome until the time of the subsequent choice (Figure 6C).

ACC inhibition reduced the influence of the state transition (common or rare) on the subsequent choice ($p = 0.007$, Bonferroni corrected for comparison of three predictors, stimulation-by-group interaction $p = 0.029$, permutation test) (Figures 6D and S5A). Stimulation did not affect how either the trial outcome ($p = 0.94$, uncorrected) or the transition-outcome interaction ($p = 0.90$, uncorrected) influenced the subsequent choice. As the transition predictor most strongly differentiates model-based and model-free strategies (Figure 2), this selective effect is consistent with disrupted model-based control. If this interpretation is correct, the effect should be stronger in those subjects that rely more on model-based strategies. This was indeed the case; the inhibition effect on the transition predictor strongly correlated across subjects with the strength of model-based influence on their choices (Figure 6E; $R = -0.91$, $p = 0.0001$), as

CellPress
OPEN ACCESS

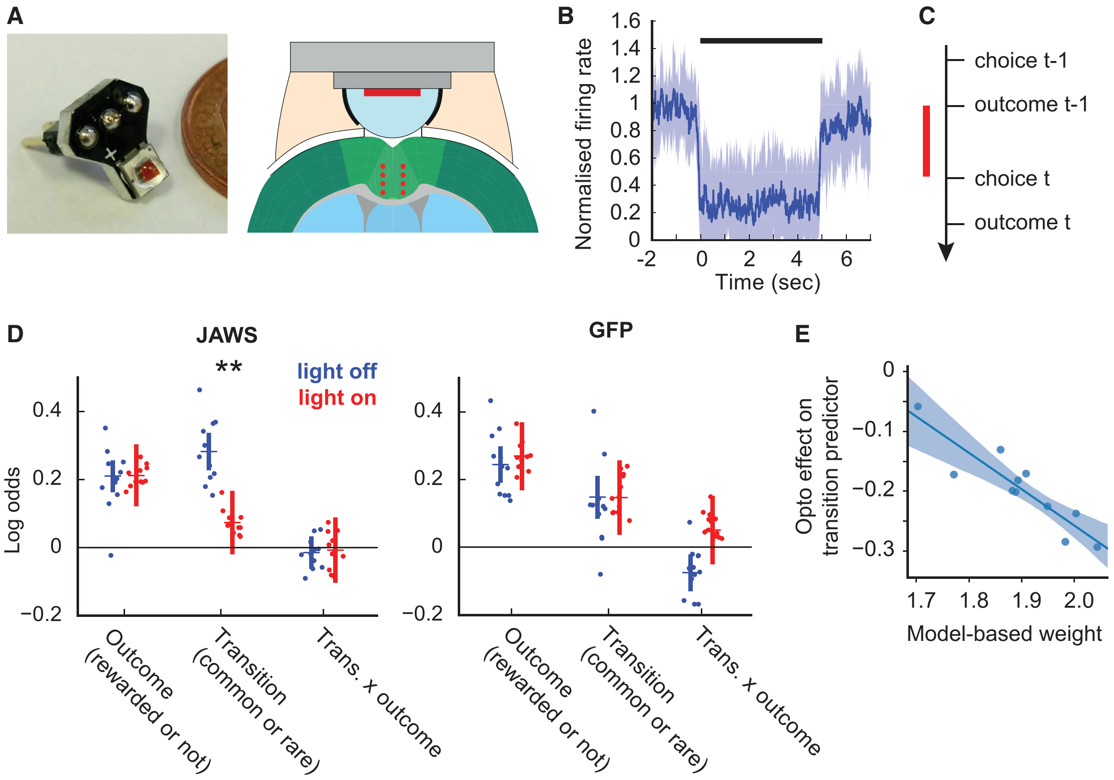

Figure 6. Optogenetic Inhibition of ACC in the Two-Step Task

(A) LED implant (left) and diagram showing implant mounted on head (right); red dots on diagram indicate location of virus injections.

(B) Normalized firing rate for significantly inhibited cells over 5 s illumination; dark blue line, median; shaded area, 25th to 75th percentiles.

(C) Timing of stimulation relative to trial events. Stimulation was delivered from trial outcome to subsequent choice.

(D) Logistic regression analysis of ACC inhibition data showing loadings for the outcome, transition, and transition-outcome interaction predictors for choices made on stimulated (red) and non-stimulated (blue) trials. **Bonferroni-corrected $p < 0.01$ between stimulated and non-stimulated trials. Error bars indicate 95% confidence intervals on the population mean, dots indicate maximum a posteriori (MAP) subject fits.

(E) Correlation across subjects between the strength of model-based influence on choice (assessed using the RL model's model-based weight parameter, G_{mb}) and the effect of optogenetic inhibition on the logistic regression model's transition predictor.

assessed by fitting the RL model to subject's behavior in the inhibition sessions using a single set of parameters for all trials.

To further test the specificity of this association, we predicted the strength of opto effect across subjects using a linear regression with a set of parameters from the RL model as predictors: the model-based weight (G_{mb}), model-free weight (G_{mf}), motor-level model-free weight (G_{mo}), and motor-perseveration strength (P_m). Model-based weight predicted the strength of opto effect on the transition predictor ($p = 0.03$), but none of the other parameters did ($p > 0.45$). These data, and an additional analysis further ruling out motor-level effects (Figure S7B), support the interpretation that inhibiting ACC blocked the influence of the state-transition on subsequent choice by disrupting model-based RL.

In both experimental and control groups, light stimulation produced a bias toward the top poke, potentially reflecting an orienting response (bias predictor $p < 0.001$, uncorrected). Reaction times were not affected by light in either group (paired t test, $p > 0.36$).

If ACC causally mediates model-based but not model-free RL, inhibiting ACC in a task in which these strategies give similar recommendations should have little effect. To test this, we performed the same ACC manipulation in a probabilistic reversal learning task, in which model-based and model-free RL are expected to generate qualitatively similar behavior (n = 10 JAWS mice, 202 sessions, 78,041 trials, n = 10 GFP mice, 202 sessions, 67,009 trials; Figure S8). Inhibiting ACC from trial outcome to subsequent choice produced a very subtle (but significant) reduction in the influence of the most recent outcome on the subsequent choice (Figure S8D; permutation test $p = 0.024$, Bonferroni corrected for six predictors, stimulation-by-group interaction $p = 0.014$). Directly comparing effect sizes between the two tasks is challenging, because in the structurally simpler reversal learning task, subjects adapt much faster to reversals (Figures 1E and S8C) and hence recent trials have a stronger influence on choices. However, the small effect in the reversal learning task relative to the influence of previous outcome on non-stimulated trials, suggests that in this simpler task, in which model-based and model-free RL both recommend repeating rewarded choices, other regions could largely compensate for ACC inhibition.

DISCUSSION

We developed a novel two-step decision task for mice that disambiguates state predictions from reward predictions in neural activity and model-based from model-free control in behavior. Calcium imaging indicated that ACC represented a set of variables required for model-based control: the state-action space of the task, the current configuration of transition probabilities linking actions to states, predicted future states given chosen actions, and whether state transitions matched these predictions. Consistent with these findings, optogenetic inhibition of ACC on individual trials reduced the influence of action-state transitions on subsequent choice, without affecting the direct reinforcing effect of reward. The strength of this inhibition effect strongly correlated across subjects with their use of model-based RL. These data suggest that the ACC is a critical controller of model-based strategies and, more specifically, reveal that the ACC is involved in predicting future states given chosen actions.

We focused on the boundary between anterior cingulate regions 24a and 24b and mid-cingulate regions 24a′ and 24b′ (Vogt and Paxinos, 2014). Though it has not to our knowledge been studied in the context of distinguishing flexible and automatic behaviors, there are anatomical and physiological reasons for considering a role for this region in model-based control. First, neurons in rat (Sul et al., 2010) and monkey (Ito et al., 2003; Matsumoto et al., 2003; Kennerley et al., 2011; Cai and Padoa-Schioppa, 2012) ACC carry information about chosen actions, reward, action values, and prediction errors during decision-making tasks. Where reward type (juice flavor) and size were varied independently (Cai and Padoa-Schioppa, 2012), a subset of ACC neurons encoded the chosen reward type rather than the reward value, consistent with a role in learning action-state relationships. In a probabilistic decision-making task in which reward probabilities changed in blocks, neuronal representations in rat ACC underwent abrupt changes when subjects detected a possible block transition (Karlsson et al., 2012). This suggests that the ACC may represent the block structure of the task, a form of world model, albeit based on learning about latent states of the world (Gershman and Niv, 2010; Akam et al., 2015), rather than the forward action-state transition model of classical model-based RL.

Second, neuroimaging in the original two-step task has identified representation of model-based value in anterior and mid-cingulate regions, suggesting that this is an important node in the model-based controller (Daw et al., 2011; Doll et al., 2015; Huang et al., 2020). Neuroimaging in a two-step task variant also found evidence for state prediction errors in dorsal ACC (Lockwood et al., 2019), consistent with our finding that ACC represented whether state transitions were common or rare. Relatedly, neuroimaging in a saccade task found ACC activation when subjects updated an internal model of where targets were likely to appear, (O'Reilly et al., 2013).

Third, ACC lesions in macaques produce deficits in tasks that require learning of action-outcome relationships (Hadland et al., 2003; Kennerley et al., 2006; Rudebeck et al., 2008), though the designs do not identify whether it is representation of the value or other dimensions of the outcome that were disrupted. Lesions of rodent ACC produce selective deficits in cost-benefit decision making in which subjects must weigh up effort against reward size (Walton et al., 2003; Rudebeck et al., 2006); however, again, the associative structures concerned are not clear.

Finally, the region of ACC we targeted provides a massive innervation to the posterior dorsomedial striatum (Oh et al., 2014; Hintiryan et al., 2016), a region necessary for learning and expression of goal-directed action as assessed by outcome devaluation (Yin et al., 2005a, 2005b; Hilario et al., 2012). Our study specifically tests the hypothesized role of ACC suggested by this body of work, showing that ACC neurons represent variables critical for model-based RL and that ACC activity is necessary for using action-state transitions to guide subsequent choice.

Our finding that different populations of ACC neurons represented reward in different states contrasts with studies in rat (Sul et al., 2010) and monkey (Seo and Lee, 2007, 2009) demonstrating that substantially more ACC neurons show a main effect of reward than a reward-choice interaction, indicating that many neurons encoded reward independent of where it was obtained (in these studies choice and reward location were fully confounded). One reason for this difference may be that Sul et al. (2010) recordings in the rat were substantially more rostral than ours. Rodent rostral circulate is more densely interconnected with frontal regions involved in reward processing, including prelimbic, infralimbic, and orbital cortices and amygdala (Fillinger et al., 2017, 2018). However, the recording location in Seo and Lee (2007, 2009) appears broadly homologous with that in our study (van Heukelum et al., 2020). Another possible reason is the tasks used, though as reward location is relevant to future choice in both, it is not obvious why reward representations should be different.

Our findings that ACC represents predictions of future states and surprise signals when those predictions are violated extends previous findings implicating ACC in prediction and surprise (Alexander and Brown, 2011; Heilbronner and Hayden, 2016). ACC neurons represent values (i.e., predictions of future reward) and reward prediction errors (Matsumoto et al., 2007; Seo and Lee, 2007; Kennerley et al., 2011). Additionally, neurons in primate medial prefrontal cortex (mPFC) respond when the animal must switch from a previously anticipated or preferred course of action (Shima et al., 1996; Isoda and Hikosaka, 2007; Seo et al., 2014). This raises the question of whether the surprise signal we see after a rare state transition reflects the state prediction error itself or its consequences for motor action. As we did not inhibit ACC at the time of the state transition, our manipulation data speak only indirectly to this. However, inhibiting ACC from outcome to choice prevented subjects using the previous state transition to inform the choice, suggesting that ACC is involved in learning from state prediction errors to guide subsequent decisions.

Our task is one of several recent adaptations of two-step tasks for animal models (Miller et al., 2017; Dezfouli and Balleine, 2017; Hasz and Redish, 2018; Groman et al., 2019). Unlike these, we introduced a major structural change to the task: reversals in the transition probabilities mapping first-step actions to second-step states. Dynamically changing transition probabilities allow neural correlates of state prediction, and the transition

probabilities themselves, to be examined. Additionally, they prevent subjects from solving the task by inferring the current state of the reward probabilities (i.e., where rewards have recently been obtained) and learning fixed habitual strategies conditioned on this latent state (e.g., rewards on the left → choose up). This can generate behavior that looks very similar to model-based RL (Akam et al., 2015). It is a particular concern in animal two-step tasks, in which subjects are typically trained extensively, with strong contrast between good and bad options. In humans, extensive training renders apparently model-based behavior resistant to a cognitive load manipulation (Economides et al., 2015), which normally disrupts model-based control (Otto et al., 2013), suggesting that it is possible to develop automatized strategies which closely resemble planning.

It has been argued that reaction time differences following common versus rare transitions are evidence for model-based RL (Miller et al., 2017). However, when the actions necessitated by each second-step states are consistent from trial to trial, reaction time differences may reflect preparatory activity at the motor level, on the basis of correlation between first-step choice and the *action* that will be required at the second step. Indeed, recent studies in humans have demonstrated that motor responses can show sensitivity to task structure when choices are model free (Castro-Rodrigues et al., 2020; Konovalov and Krajbich, 2020). Therefore in versions of the task, including ours, that do not randomize the action associated with each second-step option from trial to trial (as done in the original human task but not in rodent versions), second-step reaction times may not provide strong evidence for model-based action evaluation.

We compared behavior on task variants with and without transition probability reversals and found that they radically change behavior. Specifically, with fixed transition probabilities, subjects were much faster to adapt to reversals in reward probability and showed no main effect of outcome on subsequent choice but a strong transition-outcome interaction (i.e., behavior looked, at least superficially, strongly model based). We suggest there are three possible interpretations of this difference in terms of RL strategy. First, it is possible that both tasks recruit model-based planning, but it has a much stronger influence on choice in the fixed task. The challenge for this account is why behavior on the two tasks is so different, as model-based RL can cope with changes in reward or transition probabilities with comparable ease. Second, apparently strongly model-based behavior with fixed transition probabilities may in fact be due to subjects' inferring the state of the reward probabilities and deploying fixed habitual actions conditioned on this, as discussed above. Third, behavior with fixed transition probabilities may be mediated by a successor representation (Dayan, 1993), which characterizes current states in terms of their likely future. Successor representations support rapid updating of values in the face of changes in the reward function (and so could generate "model-based" behavior in the fixed transition probability version), but not changes in state transition probabilities (and so could not solve the new task) (Russek et al., 2017). Both of these strategies are of substantial interest in their own right, so understanding what underpins the behavioral differences between the task variants is a pressing question for future work.

In summary, our study shows that ACC predicts which state of the world to expect given a particular choice and that ACC activity is necessary for model-based RL. More broadly, it demonstrates that mice can acquire sophisticated multi-step decision tasks quickly and effectively, bringing to bear modern genetic tools to dissect mechanisms of model-based decision making.

STAR★METHODS

Detailed methods are provided in the online version of this paper and include the following:

- KEY RESOURCES TABLE
- RESOURCE AVAILABILITY
  - Lead Contact
  - Materials Availability
  - Data and Code Availability
- EXPERIMENTAL MODEL AND SUBJECT DETAILS
- METHOD DETAILS
  - Behavior
  - Two-step task
  - Probabilistic reversal learning task
  - Optogenetic Inhibition
  - ACC imaging
- QUANTIFICATION AND STATISTICAL ANALYSIS
  - Logistic regression
  - Reinforcement learning models
  - Hierarchical modeling
  - Model comparison
  - Permutation testing
  - Bootstrap tests
  - Analysis of simulated data
  - Calcium imaging analysis
  - Regression analysis of neuronal activity
  - Neuronal trajectory analysis
  - Decoding analysis

SUPPLEMENTAL INFORMATION

Supplemental Information can be found online at https://doi.org/10.1016/j.neuron.2020.10.013.

ACKNOWLEDGMENTS

We thank Zach Mainen, Joe Patton, Mark Walton, Kevin Miller, and Bruno Miranda for discussions about the work and Tim Behrens, Nathaniel Daw, and Geoff Schoenbaum for comments on the manuscript. We acknowledge the use of the Champalimaud Scientific and Technological Platforms and the University of Oxford Advanced Research Computing (ARC) facility (https://doi.org/10.5281/zenodo.22558). T.A. was funded by the Wellcome Trust (WT096193AIA). R.M.C. was funded by the National Institutes of Health (5U19NS104649) and a European Research Council (ERC) Consolidator Grant (CoG) (617142). P.D. was funded by the Gatsby Charitable Foundation, the Max Planck Society, and the Humboldt Foundation. M.P., I.R.-V., and I.M. were funded by Fundação para a Ciência e Tecnologia (SFRH/BD/52222/2013, PD/BD/105950/2014, SFRH///2011).

AUTHOR CONTRIBUTIONS

Conceptualization, T.A., P.D., and R.M.C.; Investigation, T.A., I.R.-V., I.M., X.Z., M.P., and R.F.O.; Data Curation, T.A., I.M., M.P., and R.F.O.; Formal

Analysis, T.A.; Writing – Original Draft, T.A.; Writing – Review & Editing, T.A., P.D., and R.M.C.; Funding Acquisition, T.A. and R.M.C.; Supervision, P.D. and R.M.C.

DECLARATION OF INTERESTS

The authors declare no competing interests.

Received: May 22, 2020
Revised: September 1, 2020
Accepted: October 9, 2020
Published: November 4, 2020

REFERENCES

Adams, C.D., and Dickinson, A. (1981). Instrumental responding following reinforcer devaluation. Q. J. Exp. Psychol. Sect. B *33*, 109–121.

Akam, T., Costa, R., and Dayan, P. (2015). Simple plans or sophisticated habits? State, transition and learning interactions in the two-step task. PLoS Comput. Biol. *11*, e1004648.

Alexander, W.H., and Brown, J.W. (2011). Medial prefrontal cortex as an action-outcome predictor. Nat. Neurosci. *14*, 1338–1344.

Balleine, B.W., and Dickinson, A. (1998). Goal-directed instrumental action: contingency and incentive learning and their cortical substrates. Neuropharmacology *37*, 407–419.

Benjamini, Y., and Hochberg, Y. (1995). Controlling the false discovery rate: a practical and powerful approach to multiple testing. J. R. Stat. Soc. Ser. B Methodol. *57*, 289–300.

Cai, X., and Padoa-Schioppa, C. (2012). Neuronal encoding of subjective value in dorsal and ventral anterior cingulate cortex. J. Neurosci. *32*, 3791–3808.

Castro-Rodrigues, P., Akam, T., Snorrason, I., Camacho, M., Paixao, V., Barahona-Correa, J.B., Dayan, P., Simpson, H.B., Costa, R.M., and Oliveira-Maia, A. (2020). Explicit knowledge of task structure is the primary determinant of human model-based action. medRxiv, doi: 10.11012020.09.06.20189241.

Chuong, A.S., Miri, M.L., Busskamp, V., Matthews, G.A.C., Acker, L.C., Sørensen, A.T., Young, A., Klapoetke, N.C., Henninger, M.A., Kodandaramaiah, S.B., et al. (2014). Noninvasive optical inhibition with a red-shifted microbial rhodopsin. Nat. Neurosci. *17*, 1123–1129.

Daw, N.D., Niv, Y., and Dayan, P. (2005). Uncertainty-based competition between prefrontal and dorsolateral striatal systems for behavioral control. Nat. Neurosci. *8*, 1704–1711.

Daw, N.D., Gershman, S.J., Seymour, B., Dayan, P., and Dolan, R.J. (2011). Model-based influences on humans' choices and striatal prediction errors. Neuron *69*, 1204–1215.

Dayan, P. (1993). Improving generalization for temporal difference learning: The successor representation. Neural Computation *5*, 613–624.

Dezfouli, A., and Balleine, B.W. (2017). Learning the structure of the world: the adaptive nature of state-space and action representations in multi-stage decision-making. bioRxiv. https://doi.org/10.1101/211664.

Dolan, R.J., and Dayan, P. (2013). Goals and habits in the brain. Neuron *80*, 312–325.

Doll, B.B., Duncan, K.D., Simon, D.A., Shohamy, D., and Daw, N.D. (2015). Model-based choices involve prospective neural activity. Nat. Neurosci. *18*, 767–772.

Doll, B.B., Bath, K.G., Daw, N.D., and Frank, M.J. (2016). Variability in dopamine genes dissociates model-based and model-free reinforcement learning. J. Neurosci. *36*, 1211–1222.

Ebitz, R.B., and Hayden, B.Y. (2016). Dorsal anterior cingulate: a Rorschach test for cognitive neuroscience. Nat. Neurosci. *19*, 1278–1279.

Economides, M., Kurth-Nelson, Z., Lübbert, A., Guitart-Masip, M., and Dolan, R.J. (2015). Model-based reasoning in humans becomes automatic with training. PLoS Comput. Biol. *11*, e1004463.

Fillinger, C., Yalcin, I., Barrot, M., and Veinante, P. (2017). Afferents to anterior cingulate areas 24a and 24b and midcingulate areas 24a′ and 24b′ in the mouse. Brain Struct. Funct. *222*, 1509–1532.

Fillinger, C., Yalcin, I., Barrot, M., and Veinante, P. (2018). Efferents of anterior cingulate areas 24a and 24b and midcingulate areas 24a′ and 24b′ in the mouse. Brain Struct. Funct. *223*, 1747–1778.

Gershman, S.J., and Niv, Y. (2010). Learning latent structure: carving nature at its joints. Curr. Opin. Neurobiol. *20*, 251–256.

Ghosh, K.K., Burns, L.D., Cocker, E.D., Nimmerjahn, A., Ziv, Y., Gamal, A.E., and Schnitzer, M.J. (2011). Miniaturized integration of a fluorescence microscope. Nat. Methods *8*, 871–878.

Gillan, C.M., Kosinski, M., Whelan, R., Phelps, E.A., and Daw, N.D. (2016). Characterizing a psychiatric symptom dimension related to deficits in goal-directed control. eLife *5*, e11305.

Groman, S.M., Massi, B., Mathias, S.R., Curry, D.W., Lee, D., and Taylor, J.R. (2019). Neurochemical and behavioral dissections of decision-making in a rodent multistage task. J. Neurosci. *39*, 295–306.

Hadland, K.A., Rushworth, M.F.S., Gaffan, D., and Passingham, R.E. (2003). The anterior cingulate and reward-guided selection of actions. J. Neurophysiol. *89*, 1161–1164.

Hasz, B.M., and Redish, A.D. (2018). Deliberation and procedural automation on a two-step task for rats. Front. Integr. Neurosci. *12*, 30.

Heilbronner, S.R., and Hayden, B.Y. (2016). Dorsal anterior cingulate cortex: a bottom-up view. Annu. Rev. Neurosci. *39*, 149–170.

Hilario, M., Holloway, T., Jin, X., and Costa, R.M. (2012). Different dorsal striatum circuits mediate action discrimination and action generalization. Eur. J. Neurosci. *35*, 1105–1114.

Hintiryan, H., Foster, N.N., Bowman, I., Bay, M., Song, M.Y., Gou, L., Yamashita, S., Bienkowski, M.S., Zingg, B., Zhu, M., et al. (2016). The mouse cortico-striatal projectome. Nat. Neurosci. *19*, 1100–1114.

Huang, Y., Yaple, Z.A., and Yu, R. (2020). Goal-oriented and habitual decisions: neural signatures of model-based and model-free learning. Neuroimage *215*, 116834.

Huys, Q.J.M., Cools, R., Gölzer, M., Friedel, E., Heinz, A., Dolan, R.J., and Dayan, P. (2011). Disentangling the roles of approach, activation and valence in instrumental and pavlovian responding. PLoS Comput. Biol. *7*, e1002028.

Isoda, M., and Hikosaka, O. (2007). Switching from automatic to controlled action by monkey medial frontal cortex. Nat. Neurosci. *10*, 240–248.

Ito, S., Stuphorn, V., Brown, J.W., and Schall, J.D. (2003). Performance monitoring by the anterior cingulate cortex during saccade countermanding. Science *302*, 120–122.

Karlsson, M.P., Tervo, D.G., and Karpova, A.Y. (2012). Network resets in medial prefrontal cortex mark the onset of behavioral uncertainty. Science *338*, 135–139.

Kennerley, S.W., Walton, M.E., Behrens, T.E.J., Buckley, M.J., and Rushworth, M.F.S. (2006). Optimal decision making and the anterior cingulate cortex. Nat. Neurosci. *9*, 940–947.

Kennerley, S.W., Behrens, T.E., and Wallis, J.D. (2011). Double dissociation of value computations in orbitofrontal and anterior cingulate neurons. Nat. Neurosci. *14*, 1581–1589.

Keramati, M., Dezfouli, A., and Piray, P. (2011). Speed/accuracy trade-off between the habitual and the goal-directed processes. PLoS Comput. Biol. *7*, e1002055.

Konovalov, A., and Krajbich, I. (2020). Mouse tracking reveals structure knowledge in the absence of model-based choice. Nat. Commun. *11*, 1893.

Kool, W., Cushman, F.A., and Gershman, S.J. (2016). When does model-based control pay off? PLoS Comput. Biol. *12*, e1005090.

Lee, S.W., Shimojo, S., and O'Doherty, J.P. (2014). Neural computations underlying arbitration between model-based and model-free learning. Neuron *81*, 687–699.

CellPress
OPEN ACCESS

Lockwood, P., Klein-Flugge, M., Abdurahman, A., and Crockett, M. (2019). Neural signatures of model-free learning when avoiding harm to self and other. bioRxiv. https://doi.org/10.1101/718106.

Matsumoto, K., Suzuki, W., and Tanaka, K. (2003). Neuronal correlates of goal-based motor selection in the prefrontal cortex. Science *301*, 229–232.

Matsumoto, M., Matsumoto, K., Abe, H., and Tanaka, K. (2007). Medial prefrontal cell activity signaling prediction errors of action values. Nat. Neurosci. *10*, 647–656.

Miller, K.J., Botvinick, M.M., and Brody, C.D. (2017). Dorsal hippocampus contributes to model-based planning. Nat. Neurosci. *20*, 1269–1276.

Miranda, B., Malalasekera, W.M.N., Behrens, T.E., Dayan, P., and Kennerley, S.W. (2019). Combined model-free and model-sensitive reinforcement learning in non-human primates. bioRxiv. https://doi.org/10.1101/836007.

O'Reilly, J.X., Schüffelgen, U., Cuell, S.F., Behrens, T.E., Mars, R.B., and Rushworth, M.F. (2013). Dissociable effects of surprise and model update in parietal and anterior cingulate cortex. Proc. Natl. Acad. Sci. U S A *110*, E3660–E3669.

Oh, S.W., Harris, J.A., Ng, L., Winslow, B., Cain, N., Mihalas, S., Wang, Q., Lau, C., Kuan, L., Henry, A.M., et al. (2014). A mesoscale connectome of the mouse brain. Nature *508*, 207–214.

Otto, A.R., Gershman, S.J., Markman, A.B., and Daw, N.D. (2013). The curse of planning: dissecting multiple reinforcement-learning systems by taxing the central executive. Psychol. Sci. *24*, 751–761.

Pachitariu, M., Steinmetz, N., Kadir, S., Carandini, M., and Harris, K.D. (2016). Kilosort: realtime spike-sorting for extracellular electrophysiology with hundreds of channels. bioRxiv. https://doi.org/10.1101/061481.

Rudebeck, P.H., Walton, M.E., Smyth, A.N., Bannerman, D.M., and Rushworth, M.F.S. (2006). Separate neural pathways process different decision costs. Nat. Neurosci. *9*, 1161–1168.

Rudebeck, P.H., Behrens, T.E., Kennerley, S.W., Baxter, M.G., Buckley, M.J., Walton, M.E., and Rushworth, M.F.S. (2008). Frontal cortex subregions play distinct roles in choices between actions and stimuli. J. Neurosci. *28*, 13775–13785.

Rushworth, M.F.S., and Behrens, T.E.J. (2008). Choice, uncertainty and value in prefrontal and cingulate cortex. Nat. Neurosci. *11*, 389–397.

Russek, E.M., Momennejad, I., Botvinick, M.M., Gershman, S.J., and Daw, N.D. (2017). Predictive representations can link model-based reinforcement learning to model-free mechanisms. PLoS Comput. Biol. *13*, e1005768.

Sebold, M., Deserno, L., Nebe, S., Schad, D.J., Garbusow, M., Hägele, C., Keller, J., Jünger, E., Kathmann, N., Smolka, M.N., et al. (2014). Model-based and model-free decisions in alcohol dependence. Neuropsychobiology *70*, 122–131.

Seo, H., and Lee, D. (2007). Temporal filtering of reward signals in the dorsal anterior cingulate cortex during a mixed-strategy game. J. Neurosci. *27*, 8366–8377.

Seo, H., and Lee, D. (2009). Behavioral and neural changes after gains and losses of conditioned reinforcers. J. Neurosci. *29*, 3627–3641.

Seo, H., Cai, X., Donahue, C.H., and Lee, D. (2014). Neural correlates of strategic reasoning during competitive games. Science *346*, 340–343.

Shima, K., Mushiake, H., Saito, N., and Tanji, J. (1996). Role for cells in the presupplementary motor area in updating motor plans. Proc. Natl. Acad. Sci. U S A *93*, 8694–8698.

Smittenaar, P., FitzGerald, T.H.B., Romei, V., Wright, N.D., and Dolan, R.J. (2013). Disruption of dorsolateral prefrontal cortex decreases model-based in favor of model-free control in humans. Neuron *80*, 914–919.

Sul, J.H., Kim, H., Huh, N., Lee, D., and Jung, M.W. (2010). Distinct roles of rodent orbitofrontal and medial prefrontal cortex in decision making. Neuron *66*, 449–460.

Sutton, R.S., and Barto, A.G. (1998). Reinforcement Learning: An Introduction (Cambridge, MA: MIT Press).

van Heukelum, S., Mars, R.B., Guthrie, M., Buitelaar, J.K., Beckmann, C.F., Tiesinga, P.H.E., Vogt, B.A., Glennon, J.C., and Havenith, M.N. (2020). Where is cingulate cortex? A cross-species view. Trends Neurosci. *43*, 285–299.

Vogt, B.A., and Paxinos, G. (2014). Cytoarchitecture of mouse and rat cingulate cortex with human homologies. Brain Struct. Funct. *219*, 185–192.

Voon, V., Derbyshire, K., Rück, C., Irvine, M.A., Worbe, Y., Enander, J., Schreiber, L.R.N., Gillan, C., Fineberg, N.A., Sahakian, B.J., et al. (2015). Disorders of compulsivity: a common bias towards learning habits. Mol. Psychiatry *20*, 345–352.

Walton, M.E., Bannerman, D.M., Alterescu, K., and Rushworth, M.F.S. (2003). Functional specialization within medial frontal cortex of the anterior cingulate for evaluating effort-related decisions. J. Neurosci. *23*, 6475–6479.

Wunderlich, K., Smittenaar, P., and Dolan, R.J. (2012). Dopamine enhances model-based over model-free choice behavior. Neuron *75*, 418–424.

Yin, H.H., Knowlton, B.J., and Balleine, B.W. (2005a). Blockade of NMDA receptors in the dorsomedial striatum prevents action-outcome learning in instrumental conditioning. Eur. J. Neurosci. *22*, 505–512.

Yin, H.H., Ostlund, S.B., Knowlton, B.J., and Balleine, B.W. (2005b). The role of the dorsomedial striatum in instrumental conditioning. Eur. J. Neurosci. *22*, 513–523.

Zhou, P., Resendez, S.L., Rodriguez-Romaguera, J., Jimenez, J.C., Neufeld, S.Q., Giovannucci, A., Friedrich, J., Pnevmatikakis, E.A., Stuber, G.D., Hen, R., et al. (2018). Efficient and accurate extraction of in vivo calcium signals from microendoscopic video data. eLife *7*, e28728.

STAR★METHODS

KEY RESOURCES TABLE

REAGENT or RESOURCE	SOURCE	IDENTIFIER
Bacterial and Virus Strains		
AAV5-CamKII-Jaws-KGC-GFP-ER2	UNC vector core	Addgene #65015
AAV5-CaMKII-GFP	UNC vector core	Addgene #64545
AAV5-αCaMKII-GCaMP6f-WPRE-SV40	Penn Vector Core	Addgene #100834-AAV5
Deposited Data		
Behavioral and imaging data	This paper	https://osf.io/8jwhm/
Experimental Models: Organisms/Strains		
C57BL6 mice	Champalimaud Center vivarium	N/A
Software and Algorithms		
Python 3	Python Software Foundation	https://www.python.org/; RRID: SCR_008394
Custom analysis code	This paper	https://github.com/ThomasAkam/Two-step_ACC
pyControl	pyControl developers	https://pycontrol.readthedocs.io

RESOURCE AVAILABILITY

Lead Contact
Requests for information should be directed to the lead contact, Thomas Akam (thomas.akam@psy.ox.ac.uk).

Materials Availability
This study did not generate new unique reagents.

Data and Code Availability
Task definition and analysis code, including scripts to generate the manuscript figures are available at Github: https://github.com/ThomasAkam/Two-step_ACC. Behavioral and imaging data are available at Open Science Framework: https://osf.io/8jwhm/.

EXPERIMENTAL MODEL AND SUBJECT DETAILS

All procedures were reviewed and performed in accordance with the Champalimaud Centre for the Unknown Ethics Committee guidelines. 65 male C57BL mice aged between 2 – 3 months at the start of experiments were used in the study. Animals were housed under a 12 hours light/dark cycle with experiments performed during the light cycle. 17 subjects were used in the two-step task baseline behavior dataset. 4 subjects were used in the ACC imaging. 2 subjects were used for electrophysiology controls for the optogenetics. 14 subjects (8 JAWS, 6 GFP controls) were used for the two-step task ACC manipulation only. 14 subjects (8 JAWS, 6 GFP controls) were used for the probabilistic reversal learning task ACC manipulation only. 14 subjects (8 JAWS, 6 GFP controls) were first trained and tested on the two-step ACC manipulation, then retrained for a week on the probabilistic reversal learning task and tested on the ACC manipulation in this task. 7 JAWS-GFP animals were excluded from the study due to poor or mis-located JAWS expression. In the group that was tested on both tasks, 1 Jaws and 2 control animals were lost from the study before optogenetic manipulation on the probabilistic reversal learning task due to failure of the LED implants. The resulting group sizes for the optogenetic manipulation experiments were as reported in the results section.

METHOD DETAILS

Behavior
Mice were placed on water restriction 48 hours before the first behavioral training session, and given 1 hour *ad libitum* access to water in their home cage 24 hours before the first training session. Mice received 1 training session per day of duration 1.5 – 2 hours, and were trained 6 days per week with 1 hour *ad libitum* water access in their home cage on their day off. During behavioral training mice had access to dry chow in the testing apparatus as we found this increased the number of trials performed and amount of water

consumed. On days when mice were trained they typically received all their water in the task (typically 0.5-1.25ml), but additional water was provided as required to maintain a body weight > 85% of their pre-restriction weight. Under this protocol, bodyweight typically dropped to ~90% of pre-restriction level in the first week of training, then gradually increased over weeks to reach a steady state of ~95%–105% pre-restriction body weight.

Behavioral experiments were performed in 14 custom made 12x12cm operant chambers using pyControl (http://pycontrol.readthedocs.io/en/latest/), a behavioral experiment control system built around the Micropython microcontroller. pyControl task definition files are included in the GitHub repository.

Two-step task

The apparatus, trial structure and block structure of the two-step task are described in the results section and Figure 1. Block transitions were triggered based on subject's behavior, occurring 20 trials after an exponential moving average (tau = 8 trials) of subject's choices crossed a 75% correct threshold. The 20 trial delay between the threshold crossing and block transition allowed subjects performance at the end of blocks to be assessed without selection bias due to the block transition rule. In neutral blocks where there was no correct choice, block transitions occurred with 0.1 probability on each trial after the 40th, giving a mean neutral block length of 50 trials. Transitions from non-neutral blocks occurred with equal probability (25%) to either to another non-neutral block via reversal in the reward or transition probabilities, or to one of the two neutral blocks. Transition from neutral blocks occurred via a change in the reward probabilities only to one of the non-neutral blocks with the same transition probabilities.

Subjects encountered the full trial structure from the first day of training. The only task parameters that were changed over the course of training were the reward and state transition probabilities and the reward sizes. These were changed to gradually increase task difficulty over days of training, with this typical trajectory of parameter changes shown in Table 1. Subjects started each session with the reward and transition probabilities in the same state that the previous session finished on.

Probabilistic reversal learning task

We assessed the effects of the same ACC manipulation used in the two-step task on a probabilistic reversal learning task. In this task both model-free and model-based RL are expected to generate qualitatively similar influence of trial events on subsequent choice, i.e., rewarded choices will be reinforced, though there may be quantitative differences if the model-based system is able to learn the block structure and infer block transitions rather than relying on TD value updates.

Subjects initiated trials in a central nose-poke port, which was flanked by left and right poke ports (Figure S8A). Trial initiation caused the left and right pokes to light up, subjects then chose between them for the chance of obtaining a water reward. Reward probabilities changed in blocks, with three block types; *left good* (left = 0.75/right = 0.25), *neutral* (0.5/0.5) and *right good* (0.25/0.75). Block transitions from non-neutral blocks were triggered 10 trials after an exponential moving average (tau = 8 trials) crossed a 75% correct threshold. Block transitions from neutral blocks occurred with probability 0.1 on each trial after the 15th of the block to give an average neutral block length of 25 trials. Mice tracked the correct option (Figures S8B and S8C), choosing correctly at the ends of blocks with probability 0.80 ± 0.04 (mean ± SD), and adapting to reversals with a time constant of 3.57 trials (exponential fit tau). We assessed how previous trials affected the current choice using a logistic regression analysis with previous choices and outcomes as predictors (Figure S8D). Both previous choices and outcomes predicted the current choice with decreasing influence at increasing lag.

Optogenetic Inhibition

Experimental animals were injected bilaterally with *AAV5-CamKII-Jaws-KGC-GFP-ER2* (UNC vector core, titer: 5.9×10^{12}) using 16 injections each of 50nL (total 800nL) spread across 4 injection tracks (2 per hemisphere) at coordinates: AP: 0, 0.5, ML: ± 0.4, DV: −1, −1.2, −1.4, −1.6mm relative to dura. Control animals were injected with *AAV5-CaMKII-GFP* (UNC vector core, titer: 2.9×10^{12}) at the same coordinates. Injections were performed at a rate of 4.6nL/5 s, using a Nanojet II (Drummond Scientific) with bevelled glass micropipettes of tip diameter 50-100um. A circular craniotomy of diameter 1.8mm was centered on AP: 0.25, ML: 0, and a high power red led (Cree XLamp XP-E2) was positioned above the craniotomy touching the dura. The LED was mounted on a custom designed insulated metal substrate PCB (Figure 6A). The LEDs were powered using a custom designed constant current LED driver. In both two-step and reversal learning tasks, on stimulation trials red light (50mW, 630nM) was delivered from when the subject entered the side poke and received the trial outcome, until the subsequent choice, up to a maximum of 6 s. Stimulation was delivered on a randomly selected 1/6 (17%) of trials, with a minimum of 2 non-stimulated trials between each stimulation trial followed by a 0.25 probability of stimulation on each subsequent trial. At the end of behavioral experiments, animals were sacrificed and perfused with paraformaldehyde (4%). The brains were sectioned in 50um coronal slices and the location of viral expression was characterized with fluorescence microscopy (Figure S6).

Two animals were injected unilaterally with the JAWS-GFP virus using the coordinates described above and implanted with the LED implant and a movable bundle of 16 tungsten micro-wires of 23 μm diameter (Innovative-Neurophysiology) to record unit activity. After 4 weeks of recovery, recording sessions were performed at 24 hour intervals and the electrode bundle was advanced by 50 um after each session, covering a depth range of 300 – 1300um from dura over the course of recordings. During recording sessions mice were free to move inside a sound attenuating chamber. Light pulses (50mW power, 5 s duration) were delivered at random intervals with a mean inter-stimulus interval of 30 s. Neural activity was acquired using a Plexon recording system running Omniplex v. 1.11.3.

The signals were digitally recorded at 40000 Hz and subsequently band-pass filtered between 200 Hz and 3000 Hz. Following filtering, spikes were detected using an amplitude threshold set at twice the standard deviation of the bandpass filtered signal. Initial sorting was performed automatically using Kilosort (Pachitariu et al., 2016). The results were refined via manual sorting based on waveform characteristics, PCA and inter-spike interval histogram. Clusters were classified as single units if well separated from noise and other units and the spike rate in the 2ms following each spike was less than 1% of the average spike rate.

ACC imaging

Mice were anaesthetized with a mix of 1%–1.5% isofluorane and oxygen (1 l.min-1), while body temperature was monitored and maintained at 33°C using a temperature controller (ATC1000, World Precision Instruments). Unilateral injection of 300 nL of AAV5-αCaMKII-GCaMP6f-WPRE-SV40 (titer: 2.43×10^{13}, Penn Vector Core) into the right Anterior Cingulate Cortex (AP: +1.0 mm; ML: +0.45mm; DV: −1.4mm) was performed using a Nanojet II Injector (Drummond Scientific, USA) at a rate of 4.6 nL per pulse, every 5 s. Injection pipette was left in place 20 min post-injection before removal. 25 minutes after injection, a 1mm diameter circular craniotomy was centered at coordinates (AP: +1.0 mm; ML: +0.55mm) and a 1mm GRIN lens (Inscopix) was implanted above the injection site at a depth of −1.2 mm ventral to the surface, and secured to the skull using cyanoacrylate (Loctite) and black dental cement (Ortho-Jet, Lang Dental USA). One 1/16-inch stainless-steel screw (Antrin miniatures) was attached to the skull to secure the cement cap that fixed the lens to the skull. Mice were then given an i.p. injection of buprenorfin (Bupaq, 0.1 mg.kg-1) and allowed to recover from anesthesia in a heating mat before returning to home cage.

Three to four weeks after surgery, mice were anaesthetized and placed in the stereotactic frame, where a miniaturized fluorescence microscope (Inscopix) attached to a magnetic baseplate (Inscopix) were lowered to the top of the implanted GRIN lens, until a sharp image of anatomical landmarks (blood vessels) and putative neurons appeared in the focal plane. Baseplate was then cemented to the original head cap, allowing to fix the set focal plane for imaging.

For image acquisition during task behavior, mice were briefly anaesthetized using a mixture of isofluorane (0.5%–1%) and oxygen (1 l.min-1) and the miniaturized microscope was attached and secured to the baseplate. This was followed by a 20-30 min period of recovery in the home cage before imaging experiments. Image acquisition (nVistaHD, Inscopix) was done at 10 Hz, with LED power set to 10%–30% (0.1-0.3 mW) with a gain of 3. Image acquisition parameters were set to the same values between sessions for each mouse.

QUANTIFICATION AND STATISTICAL ANALYSIS

All analysis of behavioral data was performed in Python 3.

Logistic regression

Binary predictors used in logistic regressions predicting subjects choices (e.g., Figures 2B and 2C) are shown in Table 2. The two-step task lagged logistic regression used predictors *Choice*, *Outcome*, *Transition* and *Transition-outcome interaction* at lags 1, 2, 3-4, 5-8, 8-12 (where lag 3-4 etc. means the sum of the individual trial predictors over the specified range of lags) and predictors *Bias: top/bottom,* and *Bias:clockwise/counter-clockwise*. The *Correct* predictors was included in the previous trial regression to prevent correlations across trials from causing spurious loading on the *Transition-outcome interaction* predictor (see Akam et. al. 2015 for discussion). It was not included in the lagged regression as here the effect of earlier trials is accounted for by the lagged predictors. For the two-step task logistic regressions, the first 20 trials after each reversal in the transition probabilities was exclude from the analysis as it is ambiguous which transitions are common and rare at this point. This resulted in ~9% of trials being excluded.

The logistic regression analysis for the probabilistic reversal learning task (Figure S8D) used predictors *Choice*, and *Outcome* at lags 1, 2, 3.

Reinforcement learning models

RL model variables and parameters are listed in Table 2.

First-step model-free action values were updated as:

$$Q_{mf}(c) \leftarrow (1-\alpha_Q)Q_{mf}(c) + \alpha_Q(\lambda r + (1-\lambda)V(s)) \quad 1$$

This combines an update due to the value $V(s)$ of the second-step state reached, with direct update of the first-step action value by the trial outcome due to eligibility traces. The relative influence of each is controlled by the eligibility trace parameter λ.

Second-step state values were updated as:

$$V(s) \leftarrow (1-\alpha_Q)V(s) + \alpha_Q r \quad 2$$

In models that included value forgetting this was implemented as:

$$Q_{mf}(c') \leftarrow (1-f_Q)Q_{mf}(c') \quad 3$$

$$V(s') \leftarrow (1 - f_Q)V(s') \quad (4)$$

Action-state transition probabilities used by the model-based system were updated as:

$$P(s|c) \leftarrow (1 - \alpha_T)P(s|c) + \alpha_T \quad (5)$$

$$P(s'|c) \leftarrow (1 - \alpha_T)P(s'|c) \quad (6)$$

In models that included transition probability forgetting this was implemented as:

$$P(s|c') \leftarrow (1 - f_T)P(s|c') + 0.5f_T \quad (7)$$

$$P(s'|c') \leftarrow (1 - f_T)P(s'|c') + 0.5f_T \quad (8)$$

At the start of each trial, model-based first step action values were calculated as:

$$Q_{mb}(c) = \sum_s P(s|c)V(s) \quad (9)$$

Models that included model-free values for first step motor actions (e.g., left→top), updated these as:

$$Q_{mo}(c, s_{t-1}) \leftarrow (1 - \alpha_Q)Q_{mo}(c, s_{t-1}) + \alpha_Q\,(\lambda r + (1 - \lambda)V(s)) \quad (10)$$

Motor level model-free value forgetting was implemented as:

$$Q_{mo}(m') \leftarrow (1 - f_Q)Q_{mo}(m') \quad (11)$$

Where m' are all motor actions not taken.

Choice perseveration was modeled using a choice history variable $\bar{c}$. In models using single trial perseveration this was:

$$\bar{c} = c_{t-1} - 0.5 \quad (12)$$

where $c_{t-1} = 1$ if previous choice is top and 0 if previous choice is bottom.

In models using multi-trial perseveration $\bar{c}$ was an exponential moving average of recent choices, updated as:

$$\bar{c} \leftarrow (1 - \alpha_c)\bar{c} + \alpha_c(c - 0.5) \quad (13)$$

where $c = 1$ if choice is top and $c = 0$ if choice is bottom.

In models which used motor-level perseveration this was modeled using variables $\overline{m}(s_{t-1})$ which were exponential moving averages of choices following trials ending in state s_{t-1}, updated as:

$$\overline{m}(s_{t-1}) \leftarrow (1 - \alpha_m)\overline{m}(s_{t-1}) + \alpha_m(c - 0.5) \quad (14)$$

Net action values were given by a weighted sum of model-free, motor-level model-free and model-based action values, biases and perseveration.

$$Q_{net}(c) = G_{mf}Q_{mf}(c) + G_{mo}Q_{mo}(c,\ s_{t-1}) + G_{mb}Q_{mb}(c) + X(c) \quad (15)$$

Where G_{mf}, G_{mo} and G_{mb} are weights controlling the influence of respectively the model-free, motor-level model-free and model-based action values, and $X(c)$ is biases and perseveration where:

$$X(top) = B_c + B_r(s_{t-1} - 0.5) + P_c\bar{c} + P_m\overline{m} \quad (16)$$

$$X(bottom) = 0 \quad (17)$$

where $s_{t-1} = 1$ if previous second step state is left and 0 if right.

Net action values determined choice probabilities via the softmax decision rule:

$$P(c) = \frac{e^{Q_{net}(c)}}{\sum_c e^{Q_{net}(c)}} \quad (18)$$

Hierarchical modeling

Both the logistic regression analyses of subjects choices, and reinforcement learning model fitting used a Bayesian hierarchical modeling framework (Huys et al., 2011), in which parameter vectors $\boldsymbol{h}_i$ for individual sessions were assumed to be drawn from

Gaussian distributions at the population level with means and variance $\boldsymbol{\theta} = \{\boldsymbol{\mu}, \boldsymbol{\Sigma}\}$. The population level prior distributions were set to their maximum likelihood estimate:

$$\boldsymbol{\theta}^{ML} = argmax_{\boldsymbol{\theta}}\{p(D|\theta) = argmax_{\boldsymbol{\theta}}\left\{\prod_i^N \int d\boldsymbol{h}_i\, p(D_i|\boldsymbol{h}_i)p(\boldsymbol{h}_i|\boldsymbol{\theta})\right\} \quad (19)$$

Optimization was performed using the Expectation-Maximization algorithm with a Laplace approximation for the E-step at the k-th iteration given by:

$$p\left(\boldsymbol{h}_i^k\middle|D_i\right) = N\left(\boldsymbol{m}_i^k, \boldsymbol{V}_i^k\right) \quad (20)$$

$$\boldsymbol{m}_i^k = argmax_{\boldsymbol{h}}\left\{p(D_i|\boldsymbol{h})p\left(\boldsymbol{h}\middle|\boldsymbol{\theta}^{k-1}\right)\right\} \quad (21)$$

Where $N(\boldsymbol{m}_i^k, \boldsymbol{V}_i^k)$ is a normal distribution with mean $\boldsymbol{m}_i^k$ given by the maximum *a posteriori* value of the session parameter vector $\boldsymbol{h}_i$ given the population level means and variance $\boldsymbol{\theta}^{k-1}$, and the covariance $\boldsymbol{V}_i^k$ given by the inverse Hessian of the likelihood around $\boldsymbol{m}_i^k$. For simplicity we assumed that the population level covariance $\boldsymbol{\Sigma}$ had zero off-diagonal terms. For the k-th M-step of the EM algorithm the population level prior distribution parameters $\boldsymbol{\theta} = \{\boldsymbol{\mu}, \boldsymbol{\Sigma}\}$ are updated as:

$$\boldsymbol{\mu}^k = \frac{1}{N}\sum_{i=1}^N \boldsymbol{m}_i^k \quad (22)$$

$$\Sigma = \frac{1}{N}\sum_{i=1}^N\left[\left(\boldsymbol{m}_i^k\right)^2 + \boldsymbol{V}_i^k\right] - \left(\boldsymbol{\mu}^k\right)^2 \quad (23)$$

Parameters were transformed before inference to enforce constraints $(0 < \{G_{mf}, G_{mo}, G_{mb}\}, 0 < \{\alpha_Q, f_Q, \lambda, \alpha_T, f_T, \alpha_c, \alpha_m\} < 1)$.

Model comparison

To compare the goodness of fit for models with different numbers of parameters we used the integrated Bayes Information Criterion (iBIC) score. The iBIC score is related to the model log likelihood $p(D|M)$ as:

$$\log p(D|M) = \int d\boldsymbol{\theta}\, p(D|\boldsymbol{\theta})p(\boldsymbol{\theta}|M) \quad (24)$$

$$\approx -\frac{1}{2}iBIC = \log p\left(D\middle|\boldsymbol{\theta}^{ML}\right) - \frac{1}{2}\left|M\right|log\left|D\right| \quad (25)$$

Where |M| is the number of fitted parameters of the prior, |D| is the number of data points (total choices made by all subjects) and iBIC is the integrated BIC score. The log data likelihood given maximum likelihood parameters for the prior $\log p(D|\boldsymbol{\theta}^{ML})$ is calculated by integrating out the individual session parameters:

$$\log p\left(D\middle|\boldsymbol{\theta}^{ML}\right) = \sum_i^N log\int d\boldsymbol{h}p(D_i|\boldsymbol{h})p\left(\boldsymbol{h}\middle|\boldsymbol{\theta}^{ML}\right) \approx \sum_i^N log\frac{1}{K}\sum_{j=1}^K p\left(D_i\middle|\boldsymbol{h}^j\right) \quad (26)$$

Where the integral is approximated as the average over K samples drawn from the prior $p(\boldsymbol{h}|\boldsymbol{\theta}^{ML})$. Bootstrap 95% confidence intervals were estimated for the iBIC scores by resampling from the population of samples drawn from the prior.

Permutation testing

Permutation testing was used to assess the significance of differences in model fits between stimulated and non-stimulated trials. The regression model was fit separately to stimulated and non-stimulated trials to give two sets of population level parameters $\boldsymbol{\theta_s} = \{\boldsymbol{\mu_s}, \boldsymbol{\Sigma_s}\}$ and $\boldsymbol{\theta_n} = \{\boldsymbol{\mu_n}, \boldsymbol{\Sigma_n}\}$, where $\boldsymbol{\theta_s}$ are the parameters for the stimulated trials and $\boldsymbol{\theta_n}$ are the parameters for the non-stimulated trials. The difference between the population level means for the stimulated and non-stimulated conditions were calculated as:

$$\Delta\boldsymbol{\mu}_{true} = \boldsymbol{\mu_s} - \boldsymbol{\mu_n} \quad (27)$$

An ensemble of $N = 5000$ permuted datasets was then created by shuffling the labels on trials such that trials were randomly assigned to the 'stimulated' and 'non-stimulated' conditions. The model was fit separately to the stimulated and non-stimulated trials for each

CellPress
OPEN ACCESS

permuted dataset and the difference between population level means in the stimulated and non-stimulated conditions was calculated for each permuted dataset i as:

$$\Delta\boldsymbol{\mu}^{i}_{perm} = \boldsymbol{\mu}^{i}_{s} - \boldsymbol{\mu}^{i}_{n} \quad (28)$$

The distribution of $\Delta\boldsymbol{\mu}_{perm}$ over the population of permuted datasets approximates the distribution under the null hypothesis that stimulation does not affect the model parameters. The P values for the observed distances $\Delta\boldsymbol{\mu}_{true}$ are then given by:

$$P = 2\min\left(\frac{\mathbf{M}}{N}, 1 - \frac{\mathbf{M}}{N}\right) \quad (29)$$

Where $\mathbf{M}$ is the number of permutations for which $\Delta\boldsymbol{\mu}^{i}_{perm} > \Delta\boldsymbol{\mu}_{true}$.

In addition to testing for a significant main effect of the stimulation we tested for significant stimulation by group interaction. We first evaluated the true difference between the effect sizes for the two groups as:

$$\Delta_{true} = \left(\boldsymbol{\mu}^{JAWS}_{s} - \boldsymbol{\mu}^{JAWS}_{n}\right) - \left(\boldsymbol{\mu}^{GFP}_{s} - \boldsymbol{\mu}^{GFP}_{n}\right) \quad (30)$$

The approximate distribution of this difference under the null hypothesis that there was no difference between the groups was evaluated by creating an ensemble of permuted datasets in which we randomly assigned subjects to the JAWS and GFP groups and the interaction P value was calculated as above.

Permutation testing was also used to assess significance differences in logistic regression model fits to the behavior of subjects run on the task variants with and without reversals in the transition probability reversals, with permuted datasets generated by permuting subjects between the two groups.

Bootstrap tests

To test whether predictor loadings for logistic regression analyses of subjects choices were significantly different from zero, bootstrap confidence intervals on the population means $\boldsymbol{\mu}$ were evaluated by generating a set of $N = 5000$ resampled datasets by sampling subjects with replacement. P values for predictor loading significantly different from zero were calculated as:

$$P = 2\min\left(\frac{\mathbf{M}}{N}, 1 - \frac{\mathbf{M}}{N}\right) \quad (31)$$

Where $\mathbf{M}$ is the number of resampled datasets for which $\boldsymbol{\mu} > 0$.

Analysis of simulated data

For analyses of data simulated from different RL agent types (Figure 2), we first fitted each agent to our baseline behavioral dataset using the hierarchical framework outlined above. The agents used were a model-free agent with eligibility traces and value forgetting (Figures 2D–2F), and a model-based agent with value and transition probability forgetting (Figures 2G–2I) and the best fitting RL model detailed in Figure S3 (Figures 2J–2L). We then simulated data (4000 sessions each of 500 trials) from each agent, drawing parameters for each session from the fitted population level distributions for that agent. We performed the logistic regression on the simulated data, using the same hierarchical framework as for the experimental data.

Calcium imaging analysis

Pre-processing

All imaging videos were pre-processed and motion corrected using custom MATLAB code, using the Mosaic API (Inscopix). Videos were spatially down sampled 4x4 and motion corrected using a 15 to 20-point specific reference area drawn for each animal (blood vessel pattern). Black pixel borders inserted during motion correction were then removed by cropping the corrected videos.

To extract calcium signals from putative single neurons, we used the MATLAB implementation of the Constrained non-negative matrix factorization – extended algorithm (CNMF-E) (Zhou et al., 2018). Putative single units were isolated from the processed imaging videos and subsequently inspected manually for quality assessment of both spatial masks and calcium time series. Isolated putative units not matching spatial masks or temporal features of neurons were discarded and not used in following analyses. All analyses used the deconvolved activity inferred by CNMF-E. For the regression and trajectory analyses the deconvolved activity was $\log_2$ transformed. Activity was aligned across trials by warping the time period between the choice and second-step port entry to match the median trial timings, activity prior to choice and after second-step port entry was not warped. Following time warping, activity was up-sampled to 20Hz and Gaussian smoothed with 50ms standard deviation. Example activity before and after alignment and smoothing are shown in Figure S4.

Regression analysis of neuronal activity

Regression analyses of population activity (Figures 3E–3H and 5) comprised a set of linear regressions each of which predicted the $\log_2$ transformed activity of one neuron at one time point relative to trial events. For each neuron-time point we calculated the coefficient of partial determination (CPD) for each predictor, i.e., how much variance of the neurons activity at that time-point was

explained by the full regression analysis that was not explained by the regression analysis if that predictor was removed. This is a measure of how much variance is uniquely explained by a predictor that cannot be explained by the other predictors. To assess how much variance of the population activity was explained by a given predictor at a given time point, we averaged the CPDs for all neurons at that time point to yield the population CPD time-courses shown in Figures 3E and 5.

We used permutation tests to assess whether the population CPDs for each predictor at each time-point were significantly larger than expected by chance: We generated an ensemble of 5000 permuted datasets by circularly shifting the predictors relative to the neural activity by a random number of trials drawn independently for each session from the range [0, N] where N is the number of trials in the session. This permutation preserves the autocorrelation across trials in both the neural activity and the predictors but randomizes the relationship between them. We calculated P values for each predictor at each time point as the fraction of permutations for which the permuted datasets had a larger CPD than the true dataset. P values for each predictor were corrected for multiple comparison across time-points using the Benjamini–Hochberg procedure (Benjamini and Hochberg, 1995).

The regression analysis in Figures 3E–3H used binary predictors coding the choice (top or bottom), second-step state (right or left) and trial outcome (rewarded or not), as well as the two-way interactions of these predictors (e.g., choice x second-step). In Figure 5A we used an additional binary predictor coding the state of the transition probabilities (*top* → *right* / *bottom* → *left* versus *top* → *left* / *bottom* → *right*), binary predictors coding the interaction of the transition probabilities with the choice and second step, and the transition on the current trial coded clockwise (e.g., top → right) versus counter-clockwise – i.e., whether the transition was common or rare. In Figure 5B we used a predictor which coded the state of the reward probabilities as −0.5, 0, 0.5 for the *left-good*, *neutral* and *right-good* states respectively, as well as the interactions of this predictor with the choice, second-step and transition on the current trial. As the subjects knowledge of the transition/reward probabilities is ambiguous in the period following block transitions where they change, these predictors were coded 0 in the 20 trials following such changes, and ± 0.5 at other times. These analyses included only sessions where we had at least 40 trials in at least two different states of the transition (Figure 5A) or reward (Figure 5B) probabilities.

In Figure 3G we evaluated the time course for two orthogonal representations of second-step state which occurred pre- and post-trial outcome. We defined unit projection vectors from the regression weights for second-step state at a time point mid-way between choice and outcome and 250ms after outcome. We then projected the regression weights for second-step state at each time point onto these two vectors to obtain time-courses for each representation. To avoid selection bias distorting the time-courses, we divided the data into odd and even trials and used the odd trials to define projection vectors that weights from the even trials were projected onto, and vice versa.

Neuronal trajectory analysis

The activity trajectories in Figure 4 were obtained by projecting the average population activity for each trial type into the low dimensional space that captured most variance between trial types, where trial type was defined by the 8 possible combinations of choice, second-step and outcome. To find this space, we calculated the average activity for each neuron for each trial type. We then averaged these across trial types to evaluate the component of activity that was not selective to different trial types. We subtracted the non-selective activity for each neuron from that neurons average activity for each individual trial type, and concatenated across trial types to generate a data matrix of shape [n neurons, n trial types * n time point] representing how activity for each neuron deviated from its cross-trial-type average in each trial type. We performed PCA on this matrix to find the space that captured the most cross-trial-type variance and then projected the average population activity trajectory for each trial type into this space to generate Figure 4.

Decoding analysis

The decoding analysis predicted location in the tasks state-action space from neuronal activity. Ten locations were defined by the time relative to trial events and the trial choice, second-step and outcome (Figure 4D). The analysis used trial aligned neuronal activity and 250ms duration time windows: *pre-choice* (starting 300ms before subjects choice), *post-choice* (centered between choice and outcome) and *post-outcome* (starting 100ms after trial outcome). Activity was averaged across the time window to give a single value for each neuron on a given visit to a location. The analysis combined activity from multiple sessions by taking a randomly selected 10 visits to each location for each session and concatenating activity vectors from like locations across sessions to give 10 population activity vectors for each location. Location was predicted from neuronal activity using multinomial logistic regression with L2 regularisation. Decoding accuracy was assessed using stratified k-fold cross validation with 10-folds, such that each training dataset contained 9 visits to each location and each test dataset the remaining visit to each location. The analysis included the 9 sessions from 3 animals with at least 10 visits to each location. As most sessions had more than 10 visits to each location (median 66 visits), the analysis was repeated 10 times using a different random selection of visits and the decoding accuracy averaged across runs.

Neuron, Volume *109*

Supplemental Information

The Anterior Cingulate Cortex Predicts Future States to Mediate Model-Based Action Selection

Thomas Akam, Ines Rodrigues-Vaz, Ivo Marcelo, Xiangyu Zhang, Michael Pereira, Rodrigo Freire Oliveira, Peter Dayan, and Rui M. Costa

Supplementary figures:

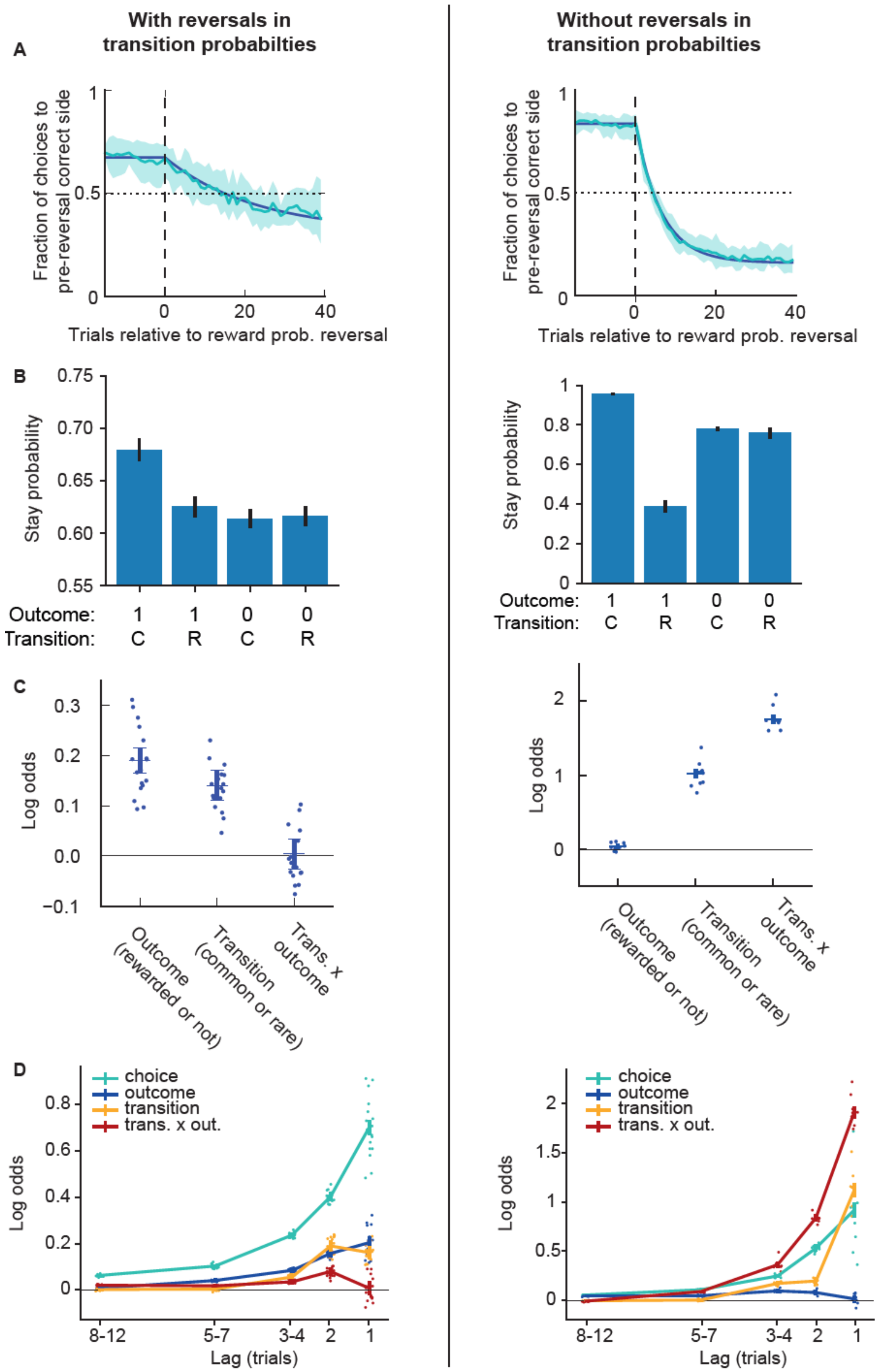

Figure S1 Behaviour without transition probability reversals, related to figures 1 & 2. The two-step task reported in the main results included reversals in the transition probability mapping the first-step actions to the second-step states, because without them, extensively trained animals could in principle learn strategies

that look like model-based RL but in fact rely on latent state inference rather than planning (Akam et al., 2015, PLOS Comp. Biol., 2015, *11*, e1004648). To assess what impact dynamically changing transition probabilities had on behaviour, we ran a version of the task where the transition probabilities linking the first step actions to second-step states were fixed (n=10 mice, 240 sessions analysed from day 22+ of training). Here we compare behaviour on the versions with transition probability reversals (left panels – reproduced from figures 1 and 2 for ease of comparison) and without transition probability reversals (right panels). The tasks were identical apart from the presence/absence of transition probability reversals. **A)** Choice probability trajectories around reward probability reversals. Pale blue line – average trajectory, dark blue line – exponential fit, shaded area – cross-subject standard deviation. Subjects were much better at tracking the correct option on the fixed task, choosing it at the end of blocks on 0.83 ± 0.04 (mean + SD) of trials, and adapting to reversals with a time constant of 6.5 trials (P<0.001 for difference between tasks on both measures, permutation test). Note that fixing the transition probabilities does not change the contrast between good and bad choices in terms of their reward probabilities. **B)** Stay probability analysis showing the fraction of trials the subject repeated the same choice following each combination of trial outcome (rewarded (1) or not (0)) and transition (common (C) or rare (R)). Error bars show cross-subject SEM. **C)** Logistic regression model fit predicting choice as a function of the previous trial's events. Predictor loadings plotted are; *outcome* (repeat choices following rewards), *transition* (repeat choices following common transitions) and *transition-outcome interaction* (repeat choices following rewarded common transition trials and non-rewarded rare transition trials). Error bars indicate 95% confidence intervals on the population mean, dots indicate maximum a posteriori (MAP) subject fits. The granular structure of behaviour was very different on the fixed task, with a very strong influence of the transition-outcome interaction on the subsequent choice (P<0.001, bootstrap test), a strong influence of the state transition (P<0.001), but no direct influence of the trial outcome (P=0.42) (between task differences at trial -1: P<0.001 for stronger loading on transition and transition-outcome interaction predictor, P=0.031 for weaker loading on outcome, permutation test). **D)** Lagged logistic regression model predicting choice as a function of events over the previous 12 trials. Predictors are as in **C**, predictor loading at lag x indicates the influence of events at trial t on choice at trial $t + x$. Overall, these data show that in the fixed task, where subjects can, in principle, learn habit-like mappings from where rewards have recently been obtained to the correct first-step action (e.g. rewards on the left →choose up), overall performance was higher and behaviour showed a strong transition-outcome interaction, which can be generated by model-based RL or latent state inference based strategies (Akam et al., 2015). The striking differences between behaviour on the fixed task and the version with transition reversals suggest that subjects do indeed solve them using different strategies.

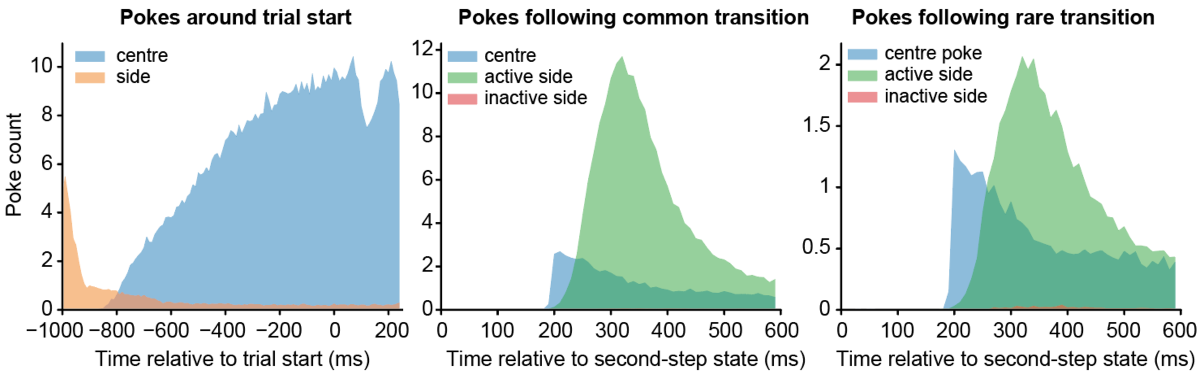

Figure S2 Poke timings around trial events, related to figure 1 A) Histogram showing the timing of pokes to the centre (i.e. top and bottom) ports (blue) and side (i.e. left and right) ports (orange) relative to the start of the trial, i.e. relative to when the centre ports become active. Subjects poke the centre ports at an increasing rate over the 1 second ITI preceding the trial start, but very rarely poke the side ports. **B)** Histogram showing the timing of pokes to the centre ports (blue) and the active (green) and inactive (red) side ports relative to the time the second-step state was entered following a common transition. The 'active' side port is the port corresponding to the second-step state reached on the trial, e.g. if the subject reached the 'left-active' state then the left port is the active side port and the right port is the inactive side port. Subjects very rarely poked the inactive side port but sometimes did an additional poke to the central ports before poking the active side. **C)** As **B** but following rare transitions.

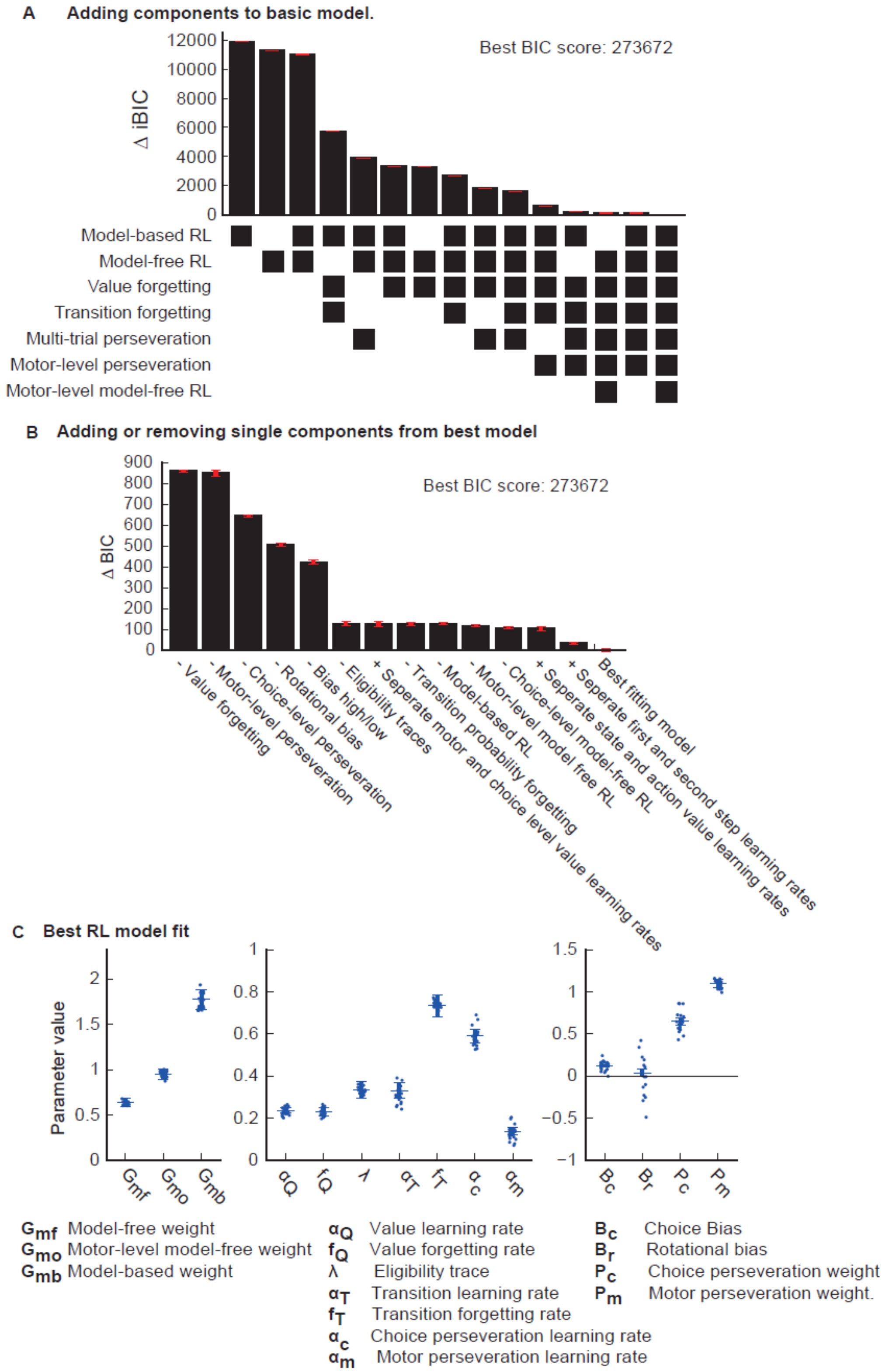

Figure S3 RL model comparison and fit, related to figure 2. A-B) RL model comparison on the baseline behavioural dataset using iBIC scores. The set of models shown in **A** were constructed by adding features to a basic model (see below). The grid below the plot indicates which features were included in each model. The set of models shown in **B** were constructed by adding or removing one feature from the best fitting model. Error-

bars indicate the bootstrap 95% confidence interval on the BIC score. **C)** Parameter values for best fitting RL model. Bars indicate 95% confidence intervals on the population mean, dots indicate maximum a posteriori (MAP) subject fits. The starting point for model comparison was the RL agent used in the original two-step task. As transition probabilities in our task are not fixed, this was modified to learn action-state transition probabilities online from experienced transitions. We also added a 'rotational' bias to move clockwise/counter-clockwise around the set of pokes (e.g. left→top, right→bottom), which we had observed in some subjects. This bias may have developed because it is the simplest fixed response pattern that was not penalised by the block transition rule (block transitions depended on behaviour, so a bias for top/bottom resulted in the preferred port spending more the time as the bad option). Using this basic model, the mixture agent incorporating both model-based and model-free RL fit better than either the pure model-free (ΔiBIC=264) or pure model-based agent (ΔiBIC=888). However, an exploratory process of model comparison indicated several additional features of the behaviour that substantially improved fit quality. The first was forgetting about actions not taken and states not visited. Value forgetting, implemented as value decay towards zero (Ito and Doya, 2009, J. Neurosci. *29*, 9861–9874), produced a dramatic improvement in fit (ΔiBIC=7698 for mixture model). Forgetting about action-state transition probabilities, implemented as decay towards a uniform distribution, further improved fit (ΔiBIC=643). The second feature found to improve fit quality was multi-trial perseveration, i.e. a tendency to repeat choices that extended over multiple trials (Akaishi et al. 2014, Neuron *81*, 195–206). Implemented as an exponential choice kernel, this provided a further substantial improvement in fit quality (ΔiBIC=1057). The final features found to improve fit were perseveration and model-free RL operating at the level of motor actions (e.g. left→top) in addition to at the level of choices (top vs bottom). Motor perseveration, implemented by maintaining separate exponential choice kernels for trials that ended on the left and right, which each influenced choices following trials ending on their respective sides, substantially improved fit quality (ΔiBIC=1503). Model-free value learning at the level of motor actions (in addition to choices) further improved goodness of fit (ΔiBIC=117). A similar finding has been reported in human two-step task behaviour where model-free value accrues to low level, task irrelevant, sensory-motor features (Shahar et al., 2019, PNAS *116*, 15871–15876). With all these additional features added to the model, the mixture agent still provided a better fit to the data than either a pure model-free (ΔiBIC=127) or pure model-based (ΔiBIC=227) agent. We tested a number of other modifications to the model including separate learning rates at the first and second step, but did not find further improvements in fit quality (see **B**). Finally, as adding features may make other features which previously improved the fit unnecessary, we tested whether removing any individual component from the model improved fit quality but again did not find further improvements (see **B**). For a more extensive discussion of the RL model-comparison, see the Supplementary Results section of the bioRxiv version of this paper (https://doi.org/10.1101/126292).

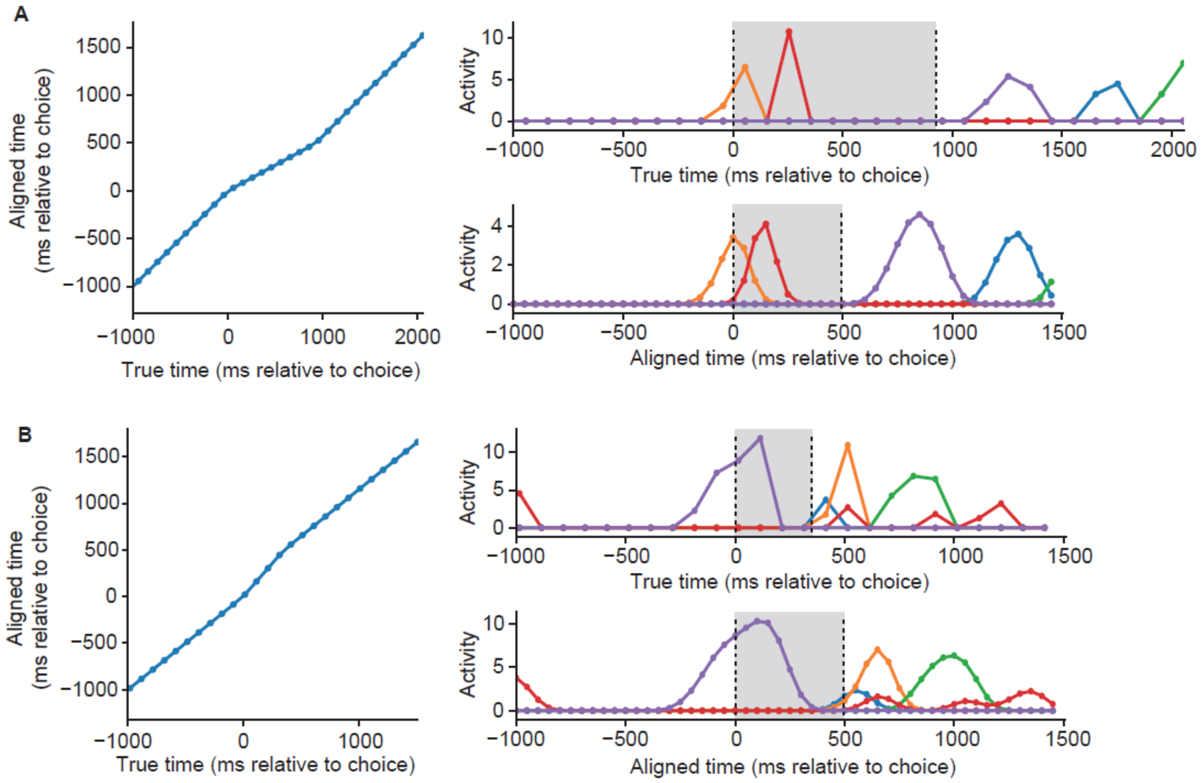

Figure S4. Calcium imaging alignment, up-sampling and smoothing, related to figure 3 & STAR methods. A) Alignment of imaging data on a trial where the interval between choice and second-step port entry was longer than the median interval. Left panel shows the true and aligned times of microscope frames plotted against each other. Right top panel shows the activity of 5 neurons before alignment. Vertical dashed lines show the times of choice and second-step port entry. Right bottom panel shows the activity of the same 5 neurons after alignment, up-sampling and smoothing. Grey shaded regions indicate the interval between choice and second-step port entry that is time-warped **B)** As for **A** but for a trial where the interval between choice and second-step port entry was shorter than the median interval.

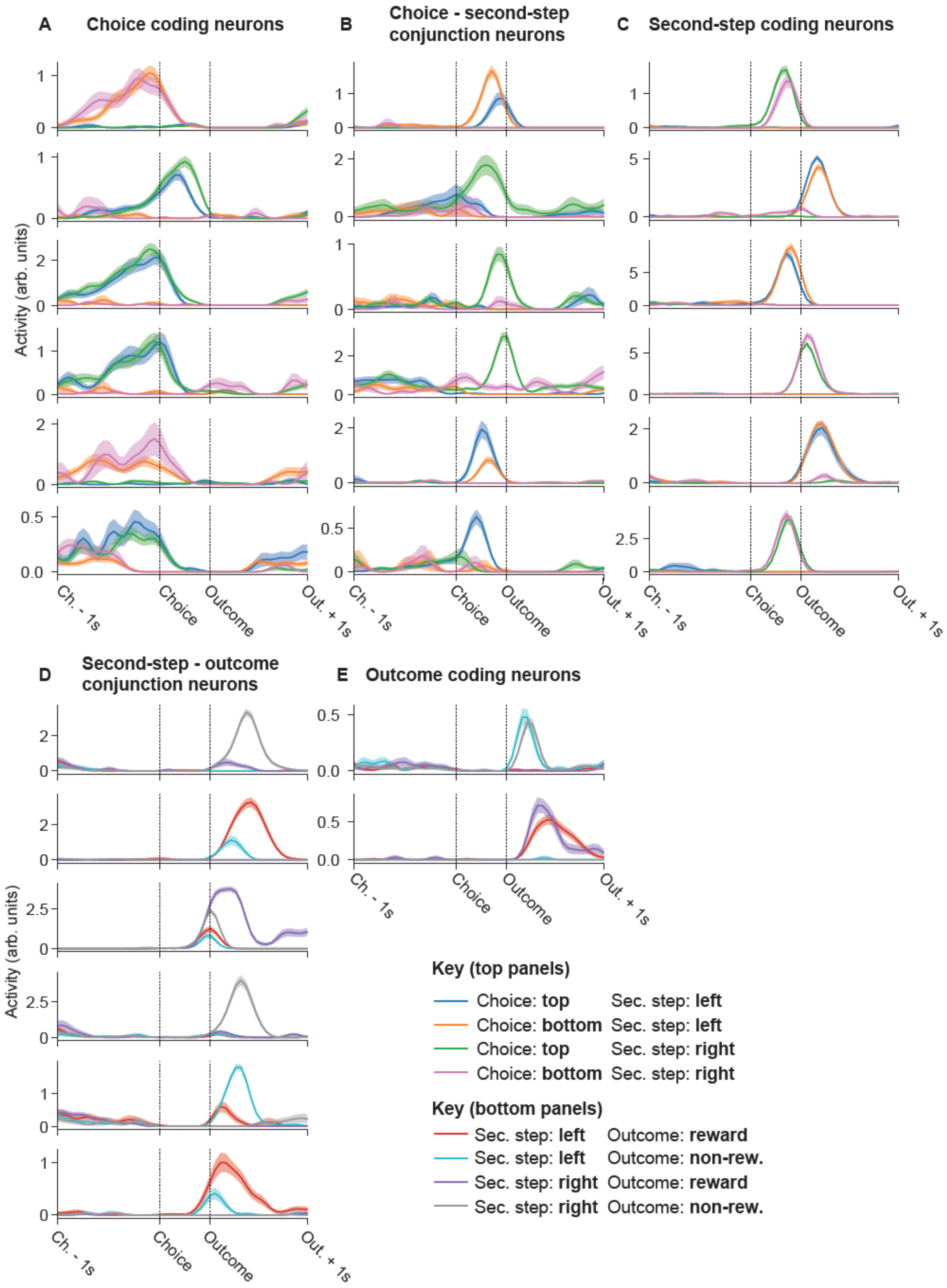

Figure S5 Example neuron activity, related to figure 3. A) Activity for six example neurons which coded the subjects choice. Traces show average activity across the trial for each of 4 conditions defined by the choice and second-step state reached, shaded areas show cross-trial standard error. **B)** As **A** but for neurons which were tuned to particular conjunctions of choice and second-step state. **C)** As **A** but for neurons which coded second-step state. **D)** As **A** but for neurons which were tuned to particular conjunctions of second-step state and outcome. Trials were split by second-step state and outcome. **E)** As **D** but for neurons which were tuned to trial

outcome. Only 2 neurons are shown due to the difficulty of finding neurons strongly tuned to outcome irrespective of second-step state.

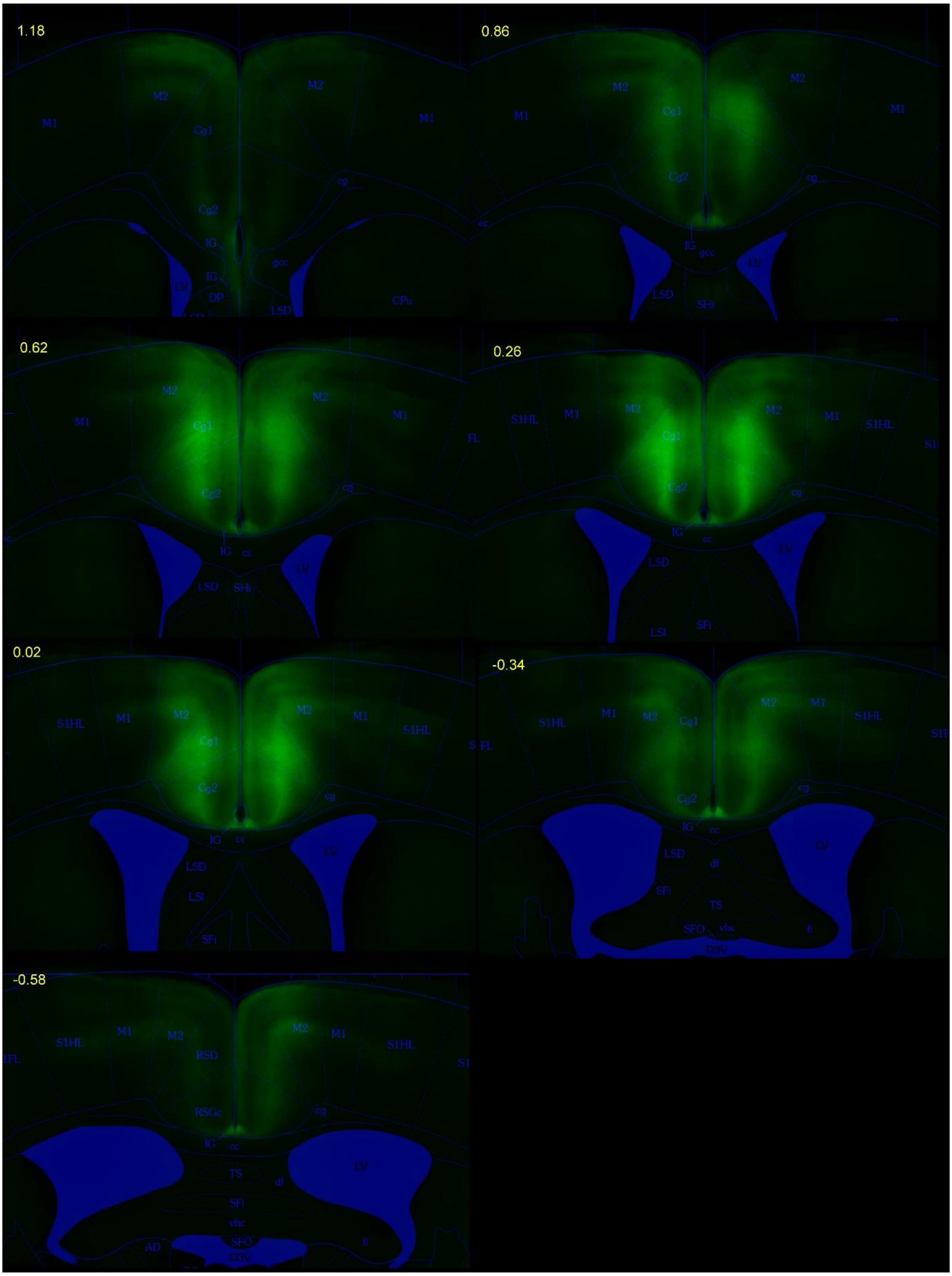

Figure S6. JAWS expression, related to figure 6. Average JAWS-GFP fluorescence for all JAWS-GFP animals included in the study aligned onto reference atlas (Paxinos and Franklin, 2007). Numbers indicate anterior-posterior position relative to bregma (mm).

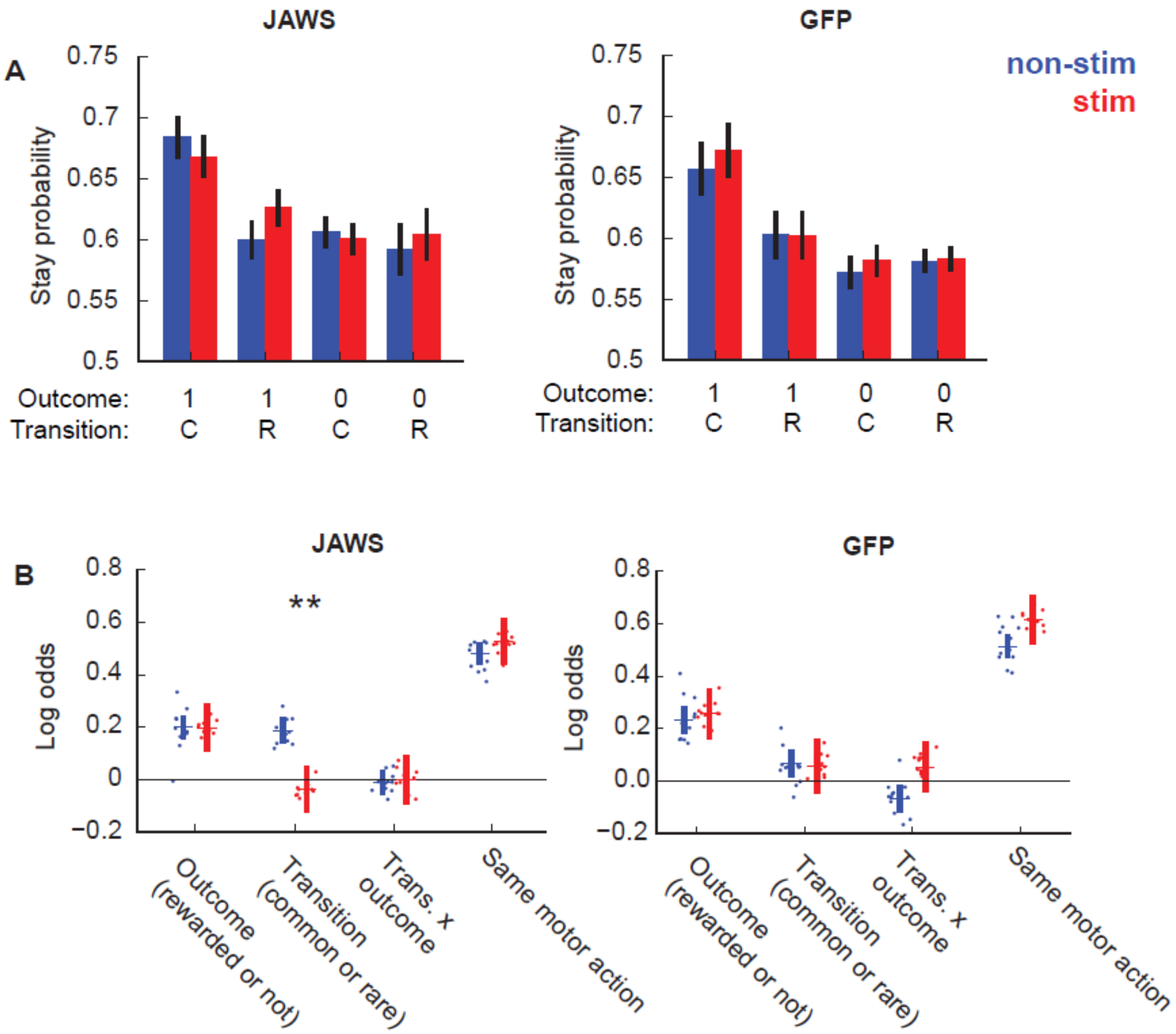

Figure S7. Optogenetic silencing of ACC in two-step task, related to figure 6. A) Stay probabilities analysis on stimulated (red) and non-stimulated (blue) trials in JAWS (top panel) and GFP (bottom panel). **B)** Evidence from RL model comparison for perseveration and model-free RL at the motor level raises a possible alternative interpretation of why ACC inhibition reduced the influence of common vs rare state transitions on choices. This is because the state transition determines which second-step state the subject ends up in, and hence the motor action required to repeat the choice on the next trial. To test whether motor-level factors can account for the ACC inhibition effect, we analysed the ACC inhibition data using a logistic regression analysis including an additional predictor *same motor action* which coded a tendency to repeat choices when this required the same motor action as the previous trial (e.g. left→top). Although *same motor action* significantly predicted repeating choice (P<0.0001, bootstrap test), ACC inhibition had no effect on the *same motor action* predictor (P=0.94 uncorrected), and the effect of ACC inhibition on the common/rare transition predictor remained significant (Bonferoni corrected P=0.0032, stim-by-group interaction P=0.032). This analysis, and the selective association between the strength of opto effect across subjects and the subjects use of model-based RL (Figure 6E), argue that the effect of ACC inhibition on sensitivity to action-state transitions is mediated by disrupted model-based RL and not motor level factors.

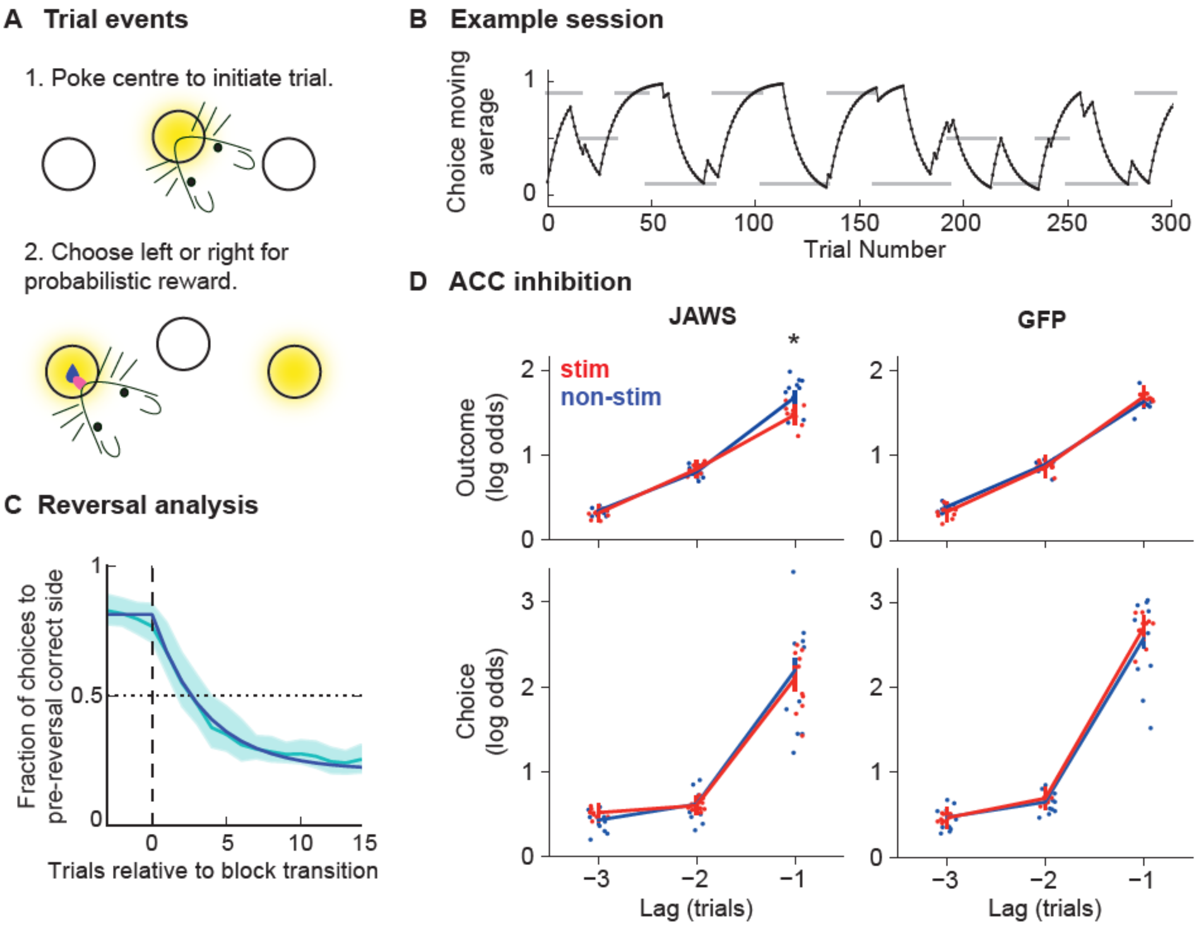

Figure S8. Optogenetic silencing of ACC in probabilistic reversal learning task, related to figure 6. A) Diagram of apparatus and trial events. **B)** Example session, black line shows exponential moving average (tau = 8 trials) of choices, grey bars indicate reward probability blocks with y position of bar indicating whether left or right side has high reward probability or a neutral block. **C)** Choice probability trajectories around reversal in reward probabilities: Pale blue line – average trajectory, dark blue line – exponential fit, shaded area – cross-subject standard deviation. **D)** Logistic regression analysis showing predictor loadings for stimulated (red) and non-stimulated (blue) trials, for the ACC JAWS (left panel) and GFP controls (right panel). Bars indicate 95% confidence intervals on the population mean, dots indicate maximum a posteriori (MAP) subject fits. * indicates significant difference (P<0.05, Bonferroni corrected for six predictors) between stimulated and non-stimulated trials.
